# Genomic profiling identifies actionable DNA-repair defects in a new cervical cancer model

**DOI:** 10.1038/s41598-026-59964-z

**Published:** 2026-07-29

**Authors:** Robert Polten, Ivana Kutle, Jan Lennart Stalp, Jens Hachenberg, Philipp John-Neek, Ann-Kathrin Seyda, Dhanya Ramachandran, Rafaela Schmidtke, Valeriia Obrizan, Lara Kokemüller, Doris Steinemann, Jonathan Lühmann, Yvonne Lisa Behrens, Gudrun Göhring, Stephan Bartels, Malte Gronewold, Lavinia Neubert, Jan C. Kamp, Dirk Schaudien, Robert Geffers, Peter Hillemanns, Thilo Dörk, Rüdiger Klapdor, Michael Morgan, Axel Schambach

**Affiliations:** 1 Institute of Experimental Hematology, Hannover Medical School, 30625 Hannover, Germany; 2 Department of Gynecology and Obstetrics, Hannover Medical School, 30625 Hannover, Germany; 3 Department of Human Genetics, Hannover Medical School, 30625 Hannover, Germany; 4 Institute of Pathology, Hannover Medical School, 30625 Hannover, Germany; 5 Biomedical Research in Endstage and Obstructive Lung Disease Hannover (BREATH), German Center for Lung Research (DZL), 30625 Hannover, Germany; 6 Department of Respiratory Medicine and Infectious Diseases, Hannover Medical School, 30625 Hannover, Germany; 7 Fraunhofer Institute for Toxicology and Experimental Medicine, ITEM, 30625 Hannover, Germany; 8 Genome Analytics, Helmholtz Center for Infection Research (HZI), Braunschweig, Germany; 9 Department of Gynecology and Obstetrics, Albertinen Hospital Hamburg, 22457 Hamburg, Germany; 10 Division of Hematology/Oncology, Boston Children’s Hospital; Department of Pediatric Oncology, Dana-Farber Cancer Institute, Harvard Medical School, Boston, MA 02115 USA

**Keywords:** Targeted therapy, DNA damage, Cervical cancer, HPV-negative, PARP inhibitor, Cancer, Computational biology and bioinformatics, Drug discovery, Genetics, Oncology

## Abstract

Genetic profiling of a new HPV-negative cervical cancer model identified pathogenic variants in genes implicated in oncogenic signaling, cell cycle regulation, and DNA damage repair pathways, including homologous recombination, non-homologous end-joining, and mismatch repair. Deficiencies in *BRCA*2, RAD51 and MLH1 were among these actionable targets. The newly described cervical cancer model revealed sensitivity towards the PARP inhibitor olaparib, which was further augmented upon combination with platinum-based chemotherapeutics. In contrast, *BRCA1/2*-proficient cervical cancer cells exhibited greater resistance to these strategies. This study highlights the potential of in-depth genetic analysis to identify genetic susceptibilities to guide personalized medicine approaches.

**Supplementary Information:**

The online version contains supplementary material available at 10.1038/s41598-026-59964-z.

## Introduction

Cervical cancer, the most prevalent gynecologic malignancy, imposes a significant global health burden, with over 600,000 new cases and more than 300,000 deaths annually^1^. Patients with advanced-stage cervical cancer face especially poor outcomes, with a five-year survival rate of less than 20%, and recurrent disease remains a complex therapeutic challenge. Cervical cancer typically arises in the “transformation zone” at the endo- and ectocervix border. The majority of cervical cancers are classified as squamous cell carcinoma (60–80%), followed by adenocarcinoma (10–20%), with adenosquamous and small cell carcinomas being less common^2^. Cervical cancer is typically associated with a persistent human papillomavirus infection (HPV), a non-enveloped DNA virus that can be detected in at least 90% of cervical cancer patients. Among the over 400 types of HPV identified, HPV16 and HPV18 were found in approximately 70% of all cervical cancer cases^3,4^.

While cervical cancers are often associated with a duplication of chromosome 3q, further common oncogenic driver mutations affect *PIK3CA*, *KRAS*, and *EGFR*^5,6^. In HPV-positive cervical cancers, the viral oncoproteins E6 and E7 are the main disease drivers. E6 and E7 target the tumor suppressors p53 and pRB, among others, leading to cell transformation and cancer progression^7^. However, the mechanisms underlying HPV-negative cervical cancer development remain poorly understood. This knowledge gap is particularly concerning considering the worse prognosis, including the significantly lower overall survival (OS) and disease-free survival (DFS) compared to HPV-positive cancers^8^. Furthermore, while HPV vaccines effectively prevent HPV-positive cervical cancers, they do not address HPV-negative cases, leaving this subgroup without equivalent prophylactic measures. Treatment of cervical cancer is usually independent of the HPV association, with surgery, chemotherapy, and radiotherapy as the current standard of care for advanced cervical cancers^9^. Depending on the clinical diagnosis, chemoradiation strategies can be supported by immune checkpoint inhibitors (ICI) such as pembrolizumab^10^ or anti-angiogenic drugs, including bevacizumab, e.g., in combination with the microtubule inhibitor paclitaxel^11^.

The use of suitable model systems is fundamental to the development of new and effective treatment options. While HPV-positive cervical cancers can be modeled by at least nine different cell lines in vitro, including the well-known HeLa cells (HPV18-positive) and SiHa cells (HPV16-positive), the availability of HPV-negative cell lines remains scarce^12^ and is currently limited primarily to only two cell lines: C33A cells that were isolated in 1964 from a squamous cell carcinoma^13^ and OMC-4 cells that were isolated in 1987 from an adenocarcinoma^14^. Therefore, novel model systems are crucial to enhance understanding and enable targeted therapeutic advancements for HPV-negative cervical cancers.

In the present study, we report the establishment and characterization of a new HPV-negative cervical cancer cell line derived from primary cervical cancer material. This highly proliferative, non-immortalized model was comprehensively analyzed, including genetic profiling, and several treatment strategies were evaluated for efficacy based on predicted pharmacogenetic susceptibilities. This work presents a valuable new resource to study this challenging subtype of cervical cancer and underlines the strengths of targeted therapies.

## Methods

### Isolation and cultivation

Primary cervical cancer tissue was surgically removed from the patient and directly transferred on ice to the laboratory for further processing. To isolate primary cervical cancer cells, the tissue was minced with a scalpel into small pieces. Cells were then dissociated by incubating the tissue pieces for 90 min in Dispase II (1 mg/mL) (Roche Diagnostics, Mannheim, Germany) at 37 °C. Dissociated cells were passed through a 70 µM filter (Sarstedt, Germany) and washed twice with PBS to avoid residual dissociation reagents. Finally, cells were plated onto 6-well adherent plates (Sarstedt, Germany) and cultivated, like SiHa and HeLa cells (ATCC, Manassas, VA, USA), in complete RPMI-1640 medium (supplemented with 10% FBS, 1% sodium pyruvate, 1% penicillin/streptomycin) (PAN-Biotech, Aidenbach, Germany). All cells were cultivated under standard humidified conditions at 37 °C and 5% CO_2_. This investigation was approved by the Hannover Medical School ethics committee (Nr. 6090_BO_K_2018).

### Immunohistochemistry staining

For immunohistochemistry (IHC), 2-µm thick sections of FFPE tissue blocks were mounted on superfrost slides (Thermo Fisher Scientific, Rockford, IL, USA). Slides were deparaffinized, rehydrated conventionally, and subjected to immunohistochemical staining using a Benchmark Ultra automated stainer (Ventana, Tucson, AZ, USA). The CC1 mild solution (Ventana) was used for antigen retrieval, and the ultraViewDAB kit (Ventana) was used for signal detection. Antibodies used for IHC (1:100) included the monoclonal antibodies P63 clone 4A4 (BIOCARE/Zytomed, Berlin, Germany), PAX8 clone MRQ-50 (Roche Diagnostics, Mannheim, Germany), P40 clone BC28 (Roche Diagnostics, Mannheim, Germany), P16 clone E6H4 (Roche Diagnostics, Mannheim, Germany), Ki67 clone 30.9 (Roche Diagnostics, Mannheim, Germany), PD-L1 clone SP263 (Roche Diagnostics, Mannheim, Germany), FAP polyclonal (Abcam, Cambridge, United Kingdom), CD3 clone 2GV6 (Roche Diagnostics, Mannheim, Germany), CD11b clone EPR1344 (Abcam, Cambridge, United Kingdom), CD19-163-L-CE (Leica, Wetzlar, Germany), CD31 clone JC70 (Roche Diagnostics, Mannheim, Germany), and CD56 clone MRQ-42 (Roche Diagnostics, Mannheim, Germany).

### Immunofluorescence imaging

For immunofluorescence staining, 3000 cells were seeded onto adherent 15-well 3D ibiTreat microscopy plates (Ibidi, Fitchburg, WI, USA) in RPMI-1640 medium and incubated overnight at 37 °C to allow cell adherence to the well surface. The next day, the medium was removed, and cells were washed once with PBS. Cells were fixed in 4% paraformaldehyde (PFA) for 20 min. Afterward, cells were washed three times to remove residual PFA and permeabilized with 0.3% Triton X-100 for 5 min. Subsequently, cells were washed with PBS, blocked with 3% bovine serum albumin (BSA) for 30 min at room temperature, and stained overnight at 4 °C with primary antibodies (1:50 in PBS); anti--Tubulin (clone B-5-1-2, Sigma-Aldrich, St. Louis, MO, USA) and anti-Vimentin (clone D21H3, Cell Signaling Technology, Danvers, MA, USA). The next day, cells were washed twice with PBS and then incubated with secondary antibodies (AF-488 anti-mouse, Cy3 anti-rabbit, Jackson ImmunoResearch Labs, West Grove, PA, USA) and DAPI (Sigma-Aldrich, St. Louis, MO, USA) for 1 h at room temperature. Finally, cells were washed twice with PBS before being covered in ProLong^TM^ Gold antifade reagent mounting media (#P36930, Invitrogen, Waltham, MA, USA). Images were acquired using the Leica SP8 confocal microscope at the core unit for laser microscopy at Hannover Medical School (MHH).

### HPV testing

The Abbott RealTime High-Risk HPV assay (Abbott, Chicago, IL, United States) was used to screen for HPV variants in the cervical smear sample based on the manufacturer’s instructions. To screen for HPV in the cervical cancer tissue and the isolated cancer cells, the Vision*Array*® Chip (#VA-0001-10, ZytoVision GmbH, Bremerhaven, Germany) was used to screen for 41 clinically relevant HPV genotypes. Additionally, WES data were systematically screened for alignment with all available FASTA sequences of the 461 human HPV types cataloged in the Papillomavirus Episteme (PaVe) database (https://pave.niaid.nih.gov; accessed January 9, 2025). Sequence alignment was conducted using the nucmer algorithm on the MHH Galaxy Server.

### Mycoplasma testing

To screen the cells for mycoplasma contamination, the genomic DNA (gDNA) was isolated with the QIAamp DNA Blood Mini Kit (Qiagen N.V., Venlo, Netherlands) based on the manufacturer’s protocol. The gDNA was amplified by polymerase chain reaction (PCR) using the Taq-DNA Polymerase (#201207, Qiagen N.V., Venlo, Netherlands) and a master mix (1X Taq-Buffer, 0.25 µM primer mix, 250 µM dNTPs, 1.25 units Taq-Polymerase, 2.5 ng internal control) and 60 ng of sample DNA. PCR amplification steps were performed according to the manufacturer’s instructions. To detect several mycoplasma strains, we used a primer mix including primers with the following forward primer sequences: 5´-CGCCTGAGTAGTACGTTCGC-3´; 5´-CGCCTGAGTAGTACGTACGC-3´; 5´-TGCCTGGGT AGTACATTCGC-3´; 5´-TGCCTGAGTAGTACATTCGC-3´; 5´-CGCCTGAGTAGTATGCTCGC-3´, 5´-CACCTGAGTAGTATGCTCGC-3´; 5´-CGCCTGGGTAGTACATTCGC-3´, and the following reverse primer sequences: 5´-GCGGTGTGTACAAGACCCGA-3´; 5´-GCGGTGTGTACAAAACCC GA-3´; 5´-GCGGTGTGTACAAACCCCGA-3´. The PCR was performed using the Biometra® TPersonal thermocycler (Biometra, Göttingen, Germany).

### Viral vector transduction for fluorescent marker expression

For the production of lentiviral vector supernatants, 5 × 10^6^ HEK-293T cells were first seeded onto 10 cm cell culture dishes. The next day, HEK-293T cells were transfected using calcium-phosphate with 25 µM chloroquine in a split-packaging design^15^. We used 5 µg LV.SFFV.mcherry.pre, 12 µg pcDNA3.GP.4xCTE, 6 µg pRSV-Rev, 2 µg pMD.G-VSVg. Viral supernatants were harvested 24 h and 48 h after initial transfection and filtered through Millex-GP 0.22 μm filters (Millipore, Schwalbach, Germany). Supernatants were concentrated by ultracentrifugation for 2 h at 76,615×*g* and then stored at -80 °C. To stably express mCherry as a fluorescent marker in CeCa-5 cells, about 50,000 cells were seeded onto a 24-well plate and then incubated overnight at 37 °C to allow cells to adhere. The next day, the medium was removed, and 3rd generation lentiviral SIN vector supernatant encoding mCherry was added to the cells in complete RPMI medium (supplemented with 4 µg/mL protamine sulfate). Cells were spinoculated by centrifugation (2000 rpm) for 1 h at 37 °C and then transferred into an incubator. After 8 h of incubation, the medium was exchanged with complete RPMI-1640 medium without protamine sulfate. Seven days later, the transduction efficacy was evaluated using a CytoFLEX S flow cytometer (Beckman Coulter, Brea, CA, USA). The bulk cells were sorted at the MHH sorting facility using the BD FACSAria™ Fusion flow cytometer to obtain an enriched population of mCherry^+^ CeCa-5 cells.

### Vector copy number determination

The genomic DNA (gDNA) of mCherry^+^ CeCa-5 cells was isolated using the QIAamp DNA Blood Mini Kit (Qiagen N.V., Venlo, Netherlands) according to the manufacturer’s protocol. To calculate the mean vector copy number (mVCN), the gDNA was analyzed by real-time quantitative polymerase chain reaction (qPCR) using the StepOnePlus device (Applied Biosystems, Foster City, CA, USA). The TaqMan Fast Advanced Master Mix (Thermo Fisher Scientific) was used alongside primers that target the polypyrimidine tract binding protein (*PTPB2*) and the woodchuck hepatitis virus posttranslational element (*WPRE*). *PTPB2* forward primer: 5´-TCTCCATTCCCTATGTTCATGC-3´; *PTPB2* reverse primer: 5´-GTTCCCGCAGAATGGTGAGGTG-3´; *PTPB2* probe: 5´-ATGTTCCTCGGACCAACTTG-3´; *WPRE* forward primer: 5´-GAGGAGTTGTGGCCCGTTGT-3´; *WPRE* reverse primer: 5´-TGACAGGTGGTGGCAATGCC-3´; *WPRE* probe: 5´-CTGTGTTTGCTGACGCAAC-3´.

### Antigen detection by flow cytometry

The expression of surface markers was evaluated using flow cytometry. Cells were treated with an FcR blocking reagent (130-059-901, Miltenyi Biotec) for 10 min at 4 °C prior to antibody staining. After washing, cells were incubated with the respective antibodies (1:100 in PBS) for 30 min at 4 °C. Subsequently, cells were washed twice in PBS, resuspended in flow cytometry buffer (PBS containing 3% FBS and 0.4% EDTA) with or without DAPI, and then measured on a CytoFLEX S (Beckman Coulter, Brea, CA, USA) or BD FACSCanto (Becton Dickinson, Franklin Lakes, NJ, USA) flow cytometer. Data were analyzed using the FlowJo software (Tree Star Inc., Ashland, OR, USA). The following antibodies were used: anti-CD44-FITC (clone IM7, BioLegend, San Diego, CA, USA), anti-CD44v6-PE (clone 2F10, R&D Systems, Minneapolis, MN, USA), anti-Mesothelin-APC (clone REA1057, Miltenyi Biotec, Bergisch Gladbach, Germany), anti-CD133-PE-Cy7 (clone 7, BioLegend, San Diego, CA, USA), anti-FAP-FITC (clone 427819, R&D Systems, Minneapolis, MN, USA), anti-EPCAM-PE (clone REA764 Miltenyi Biotec, Bergisch Gladbach, Germany), anti-CD45-APC (clone HI30, Becton Dickinson, Franklin Lakes, NJ, USA), anti-MUC1-PE-Cy7 (clone 16 A, BioLegend, San Diego, CA, USA), and anti-PD-L1-APC (clone 29E.2A3, BioLegend, San Diego, CA, USA).

### Proliferation assay

For flow cytometric assessment of cell proliferation, 50,000 or 100,000 cells/well were seeded onto a 6-well adherent plate (Sarstedt, Germany). For harvesting, cells were washed and then treated with Accutase for 5 min until detached. Cells were then centrifuged at 300×*g* for 5 min and finally resuspended in flow cytometry buffer. Cell numbers were evaluated at 0, 24, and 48 h after initial seeding using a CytoFLEX S flow cytometer (Beckman Coulter, Brea, CA, USA). Alternatively, live-cell imaging was used to calculate cell proliferation. Here, 5000 cells were seeded in 200 µL onto 96-well adherent plates (Sarstedt, Germany) and placed into the CELLCYTE X™ live-cell imaging device (San Diego, CA, United States). Images were acquired every 2 h for a total duration of 72 h. In 2D experiments, a phase-contrast mask was used to calculate the cell area as a readout for cell proliferation. To form 3D spheroids, 1000 cells were seeded in 200 µL complete RPMI onto ultra-low attachment plates (S-Bio, Hudson, NH, USA). The plates were centrifuged for 10 min at 125×*g* and then incubated for three days at 37 °C and 5% CO_2_. To assess 3D proliferation, the plates were placed into the CELLCYTE X™, and images were acquired every 4 h for 72 h. The total red fluorescence intensity derived from mCherry^+^ cells was used to calculate cell proliferation in the 3D assays.

### Immunoblotting

Cells were harvested using Accutase (PAN-Biotech, Aidenbach, Germany), centrifuged at 300×*g*, and supernatants were removed. Cell pellets were then resuspended in lysis buffer (50 mM Tris-HCl, pH 7.5, 150 mM NaCl, 100 mM NaF, 1% Triton X-100) supplemented with 1 mM Na_3_VO_4_, 1 mM DTT, and protease inhibitors (1x cOmplete™, Mini Protease Inhibitor Cocktail, Roche Diagnostics) and incubated for 20 min at 4 °C. Samples were then centrifuged for 15 min at 13,000×*g* (4 °C), the supernatants were collected, and total protein concentrations were determined via the Bradford assay (Cat. #5000006, Bio-Rad Laboratories, Hercules, CA, USA). For each sample, 25 µg of whole cell lysates were loaded and separated by SDS-PAGE and transferred to nitrocellulose membranes. Membranes were then blocked with 5% non-fat dry milk in Tris-buffered saline containing 0.1% Tween 20 (TBST). Membranes were stained by incubation with anti-MLH1 (clone 4C9C7, 1:1000, Cell Signaling Technology, Danvers, MA, USA), anti-PARP (Cell Signaling Technology, Danvers, MA, USA), and anti-GAPDH-HRP (1:10,000, GeneTex/BIOZOL, Eching, Germany) antibodies in 5% milk in TBST overnight at 4 °C. Membranes treated with non-conjugated primary antibodies were incubated with the appropriate anti-mouse-HRP or anti-rabbit-HRP secondary antibodies (1:4000, Cayman Chemical, Ann Arbor, MI, USA). Signals were visualized using the SuperSignal West Pico Substrate (Thermo Fisher Scientific, Waltham, MA, USA) and the FusionFX (PeqLab) imaging system.

### Fluorescence R-banding analysis and Fluorescence in-situ hybridization (FISH) analysis

Chromosome preparation and fluorescence-R banding were performed as previously described^16^. The karyotype was described according to the International System for Chromosome Nomenclature (ISCN, 2020). The FISH analysis was performed using a probe for the locus 3q26 (CytoCell EVI1 (MECOM) – CytoCell, Cambridge, UK) and a probe for the locus 13q14.2 (CytoCell Prenatal 13 – CytoCell, Cambridge, UK). A total of 100 interphase nuclei were analyzed per sample. The cut-off level for these probes was determined by analyzing 1000 interphase nuclei from ten healthy donors. The cut-off level was set at 2% (3q26 probe) and 12% (13q14 probe), respectively.

### Optical genome mapping

DNA was isolated from frozen cells using the Bionano Prep SP Frozen Cell Pellet DNA Isolation Protocol v2 (#30398 Rev B, Bionano Genomics Inc., San Diego, CA, United States). Afterward, DNA was quantified using the Qubit^TM^dsDNA BR Assay Kit and the Qubit 3.0 Fluorometer (Thermo Fisher Scientific, Waltham, United States). DNA was labeled using the Bionano Prep Direct Label and Stain Kit (Protocol #30206 Rev G; Bionano Genomics Inc.) and loaded onto a Saphyr G2.3 chip. Images were acquired using the Saphir device (Bionano Genomics Inc.), and the data was analyzed using the Bionano Access Server with the *de novo* pipeline (Tools Version 1.7.1, reference genome GRCh38/hg38).

### Whole exome sequencing

For whole exome sequencing (WES), genomic DNA was extracted from the CeCa-5 cell line (passage 30). Exonic sequences were enriched using the SureSelect XT Huma All Exon V6 library (Agilent Technologies, Santa Clara, CA, USA) and sequenced on a HiSeq2500 platform (Illumina Inc., San Diego, CA, USA). Raw exome sequencing data were called, de-multiplexed and aligned according to the GATK pipeline, and variants were annotated using the SnpEff tool (http://snpeff.sourceforge.net/). Variants were filtered according to their absence or low MAF < 0.005 in the NCBI SNP and/or 1000Genomes databases and according to their predicted effects. A list of variants in any gene of interest is available upon request.

### Targeted sequencing

Targeted next-generation sequencing was performed for a panel of selected genes involved in homology-directed or mismatch repair as described by Kokemüller et al. (2025)^17^. The analysis of five MMR genes (*MLH1*, *MSH2*, *MSH6*, *MUTYH*, *PMS2*) and twelve HDR genes (*BARD1*, *BRCA1*, *BRCA2*, *BRIP1*, *ERCC4*, *FANCM*, *PALB2*, *RAD51*, *RAD51B*, *RAD51C*, *RAD51D*, *SLX4*) did not reveal additional pathogenic variants beyond those identified in the whole exome sequencing. For subsequent wet-lab validation of prioritized variants in known tumor suppressor genes or oncogenes, Sanger sequencing was performed on genomic DNA extracted from three different samples from the same patient: (i) an EDTA blood sample from peripheral venous blood, (ii) a Thinprep cervical smear sample obtained before surgery for cervical cancer, and (iii) a sample from the newly established cell line CeCa-5 (passage 65). Exon-specific primers were used (Supplementary Table 1) to generate the respective PCR products using HotStar *Taq* DNA polymerase (Qiagen, Germany). Products were sequenced using the Big Dye Terminator Kit v1.1 (Applied Biosystems) and separated through capillary gel electrophoresis on a SeqStudio Genetic Analyzer (Applied Biosystems). Electropherograms were analyzed using Finch TV (Geospiza Inc.).

### Reverse transcription and detection of truncated transcripts

A novel *MLH1* variant was predicted by MaxEntScan^18^ to disrupt the canonical donor splice site (MaxEntScore 9.12->1.97). In order to confirm this in silico prediction and to determine the resulting transcripts, fragment length analysis and Sanger sequencing were performed on RT-PCR products obtained from total RNA extracted with Trizol from the CeCa-5 cells. For cDNA synthesis, 1 µg of total RNA was reverse transcribed with a NEB ProtoScript II kit (New England BioLabs, Frankfurt, Germany) according to the manufacturer’s protocol. PCR primers 5´-AATCCAAGTGAAGAATATGGG-3´and 5´-GTTTGCATTGGATATGTAACC-3´ were designed to amplify a portion of cDNA spanning exon 6–9 of *MLH1* transcript NM_000249.4. Expected product sizes were 248 bp for the reference transcript and 205 bp for the splice variant without exon 7 (ENSE00003521968). RT-PCR products were quality-checked after 2% agarose gel electrophoresis with a 1 kb+ ladder (Thermo Fisher Scientific, Waltham, USA) as size standard using GelRed and an UV-Imager (Intas, Göttingen, Germany). The identity of the splicing product was then confirmed by Sanger sequencing.

### Drug screening

The following drugs were evaluated by flow cytometry or live-cell imaging: cisplatin (Sigma-Aldrich, St. Louis, MO, USA), carboplatin (Sigma-Aldrich, St. Louis, MO, USA), paclitaxel (Sigma-Aldrich, St. Louis, MO, USA), PI3-Kα inhibitor 2 (Echelon Biosciences, Salt Lake City, UT, USA), olaparib (Cell Signaling Technology, Danvers, MA, USA). Sodium chloride (0.9%, J.T. Baker, Phillipsburg, NJ, USA) was used to dissolve cisplatin and carboplatin; DMSO (Sigma-Aldrich, St. Louis, MO, USA) was used to dissolve paclitaxel, PI3-Kα inhibitor 2, and olaparib. To investigate the effects of drug treatment by flow cytometry, 11,000 cells were seeded per well onto 48-well adherent plates (Sarstedt, Germany) and incubated overnight at 37 °C to allow cell adherence. The next day, serially diluted drugs were added either alone or in combinations. After 72 h of incubation, cells were harvested using Accutase and stained with DAPI (1:1000, Sigma-Aldrich, St. Louis, MO, USA) and Annexin V (1:20, PE, Becton Dickinson, Franklin Lakes, NJ, USA). Cells were then assessed on a CytoFLEX S flow cytometer (Beckman Coulter) and analyzed using the FlowJo software (Tree Star Inc., Ashland, OR, USA). Live cells were defined as Annexin V and DAPI double negative. To investigate drug treatments by live-cell imaging, 5000 cells were seeded per well in 96-well adherent plates (Sarstedt, Germany) and incubated overnight at 37 °C to allow cell adherence. The next day, serially diluted drugs were added either alone or in combinations. Plates were then placed into a CELLCYTE X™ live-cell imaging device (San Diego, United States). Images were acquired every 2 h for a total duration of 72 h. A phase-contrast mask was used to investigate cellular confluence at each time point.

### Statistical analysis

Statistical analysis was performed using the GraphPad Prism software (GraphPad, San Diego, CA, USA). One- or two-way ANOVA tests were performed to analyze for statistical significance, which is denoted by *p ≤ 0.05, **p ≤ 0.01, ***p ≤ 0.001. Error bars represent the standard deviation of the mean.

## Results

### Characterization of the primary cervical cancer tissue

**Fig. 1:**
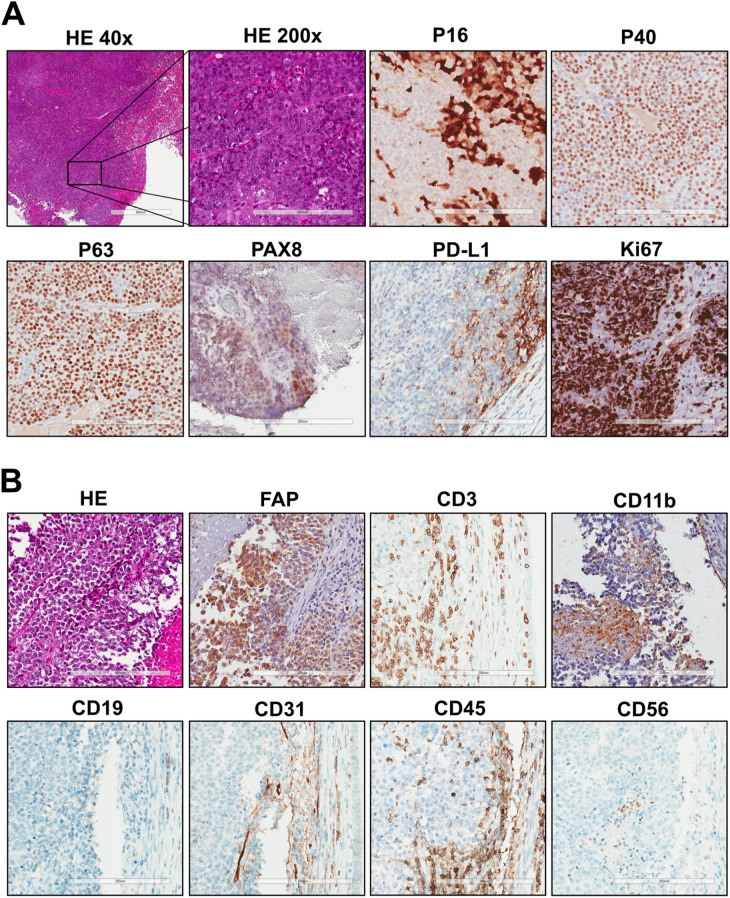
Characterization of the primary cervical cancer tissue. Immunohistochemical staining was performed on paraffin sections of the primary cervical cancer tissue. Hematoxylin and eosin (HE) stainings were performed alongside stainings for p16, p40, p63, PAX8, PD-L1, Ki67 (**A**), and for markers that identify the presence of immune cells (CD3, CD11b, CD19, CD31, CD45, CD56) and fibroblasts (FAP) (**B**). Bars correspond to 600 μm in the 40x magnification of the HE stained sample and 200 μm in all other images (200x) shown.

A tissue piece was resected during Wertheim surgery to remove the tumor from a 40-year-old patient with poorly differentiated (G3) squamous cervical cancer with lymph node metastasis (FIGO stage IIIC2). The tissue sample was further processed to characterize and classify the primary tumor by immunohistochemistry (IHC). Tumor cells were highly positive for the proliferation marker KI-67 (± 80%). The location of the tumor at the cervix, combined with positive staining for p40 and p63 but low/diffuse staining for PAX8 (± 5%) and negativity for other diagnostic markers, e.g., for CK7, indicated a non-keratinized cervical squamous cell carcinoma. The patchy p16 staining is indicative of HPV-negative cervical carcinoma. Synaptophysin, chromogranin, TTF1, and CK8/18 negativity indicated the absence of a neuroendocrine neoplasm. Also, the staining revealed a tumor proportion score (TPS) of 3% for the immune modulatory protein PD-L1, with many other markers stained negative by IHC (Fig. 1A, Supplementary Table 2).

To further characterize the primary cancer tissue, we investigated the composition of fibroblasts and immune cells in the primary cervical cancer tissue. Strong staining for fibroblast activation protein (FAP), indicative of cancer-associated fibroblasts, suggests a high abundance of fibroblasts within the primary cervical cancer tissue (Fig. 1B, Supplementary Figs. 1, 2). Immune cell analysis revealed approximately 12% positive CD11b cells (e.g., monocytes, macrophages) and less than 10% positivity for CD45 (e.g., hematopoietic cells), CD3 (e.g., T cells), CD19 (e.g., B cells), CD56 (e.g., natural killer cells) and CD31 (e.g., endothelial cells) positive cells. CD11b-positivity was mainly detected in the upper part of the depicted tumor tissue, which was identified to be cancerous due to invasive, destructive cell growth, penetration of the basal membrane, and atypical nuclei. In contrast, the non-malignant tissue (lower part of the depicted tumor tissue) revealed a low percentage of CD11b (Fig. 1B, Supplementary Fig. 1). In contrast, IHC staining revealed CD56 positivity mainly in the non-malignant tissue, while CD3, CD31, and CD45 were primarily detected at the tumor border. As most cervical cancers are associated with an HPV infection, Vision*Array*® Chip was performed as a broad screen for the presence and genotype of clinically relevant HPV in the tumor tissue. The analysis revealed the absence of any tested HPV variant (Supplementary Fig. 3A). This result was in line with results from a previous Abbott RealTime High-Risk HPV assay of a cervical smear sample taken from this patient before surgery, which was negative for HPV16, HPV18, and twelve other high-risk HPV types (31, 33, 35, 39, 45, 51, 52, 56, 58, 59, 66, 68).

### Characterization of the isolated primary cervical cancer cells

**Fig. 2:**
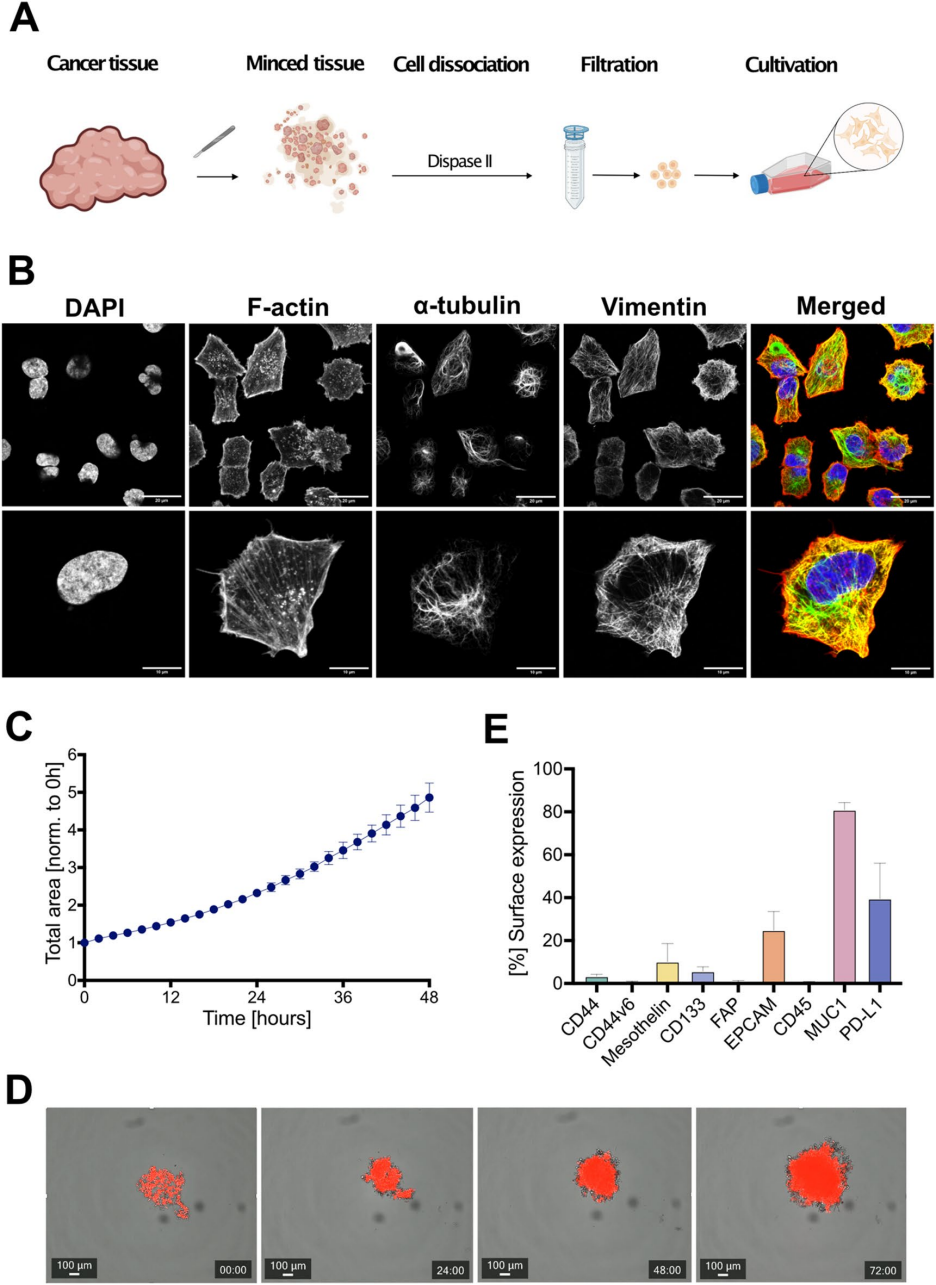
Isolation and characterization of primary cervical cancer-derived cells. Schematic overview of the isolation procedure of single cells from the primary cervical cancer tissue (**A**). Immunofluorescence staining to visualize the nucleus (DAPI, blue) and cytoskeletal elements, including microfilaments (F-actin, red), microtubules (α-tubulin, green), and intermediate filaments (Vimentin, yellow) (**B**). Scale bars correspond to 10 μm (63x magnification) and 20 μm (63x magnification, enlarged field of view). Quantification of cell proliferation that was measured via live-cell imaging for 48 h (*n = 3*) (**C**). Fluorescence images were acquired by live-cell imaging and show the 3D spheroid formation of mCherry^+^ CeCa-5 cells (**D**). Representative images from experiments accomplished in triplicate depict the formation of 3D structures at 0 h, 24 h, 48 h, and 72 h after initial seeding. Scale bar = 100 μm. Isolated primary cervical cancer cells were screened for cell surface expression of cervical cancer-associated antigens using flow cytometry (**E**). Data are displayed as mean ± SD (*n = 2–4*).

Primary cells were isolated from the patient-derived cancer tissue sample using different isolation strategies. Dispase II or Collagenase IV were used to dissociate the bulk tumor, and various media were tested to cultivate the isolated cells, including RPMI-1640, DMEM/F12, and PneumaCult. While all strategies led to the successful cultivation of cells with a cancer morphology, Collagenase IV dissociation and DMEM/F12 medium led to the growth of many cells with a fibroblast-like morphology. In contrast, the PneumaCult medium did not favor the growth of cells with a fibroblast-like morphology but also reduced the proliferation of cells with a cancer morphology. RPMI-1640 and Dispase II isolation supported the proliferation of cells with a cancer morphology but limited the proliferation of cells with a fibroblast-like morphology. Several clusters of cancer cells were isolated and used to cultivate a purer population of cancer cells and to avoid potential fibroblast contamination. Subsequently, these isolated primary cervical cancer cells were expanded in RPMI-1640 medium and cultivated long-term (passage > 175) without immortalization (Fig. 2A). These primary cells were named “CeCa-5” cells. Similar to the primary cervical cancer tissue, we investigated the HPV status of CeCa-5 cells using the Vision*Array* Chip, which also showed the absence of any tested HPV genotype in the cells derived from the primary tissue (Supplementary Fig. 3B). Mycoplasma testing illustrated the absence of mycoplasma contamination throughout the duration of CeCa-5 cell cultivation (Supplementary Fig. 4). Isolated cells were stained for cytoskeletal markers, including F-actin (microfilaments), α-tubulin (microtubules), and vimentin (intermediate filaments), revealing relatively small, homogenous, and mononucleated cells with polygonal cell morphologies (Fig. 2B). A combination of flow cytometry-based cell counting and live-cell imaging was utilized to assess the proliferative capacity of CeCa-5 cells. These assays revealed average doubling times of approximately 20 h when cultured in 2D monolayers (Fig. 2C, Supplementary Fig. 5A). To assess the proliferative capacity of CeCa-5 cells in 3D spheroid cultures, cells were transduced with a lentiviral vector to stably express mCherry (Supplementary Fig. 5B, C), which was used to quantify the total red fluorescence intensity as a readout for cell proliferation. mCherry^+^ CeCa-5 cells showed successful spheroid formation (Fig. 2D), and sustained their high cell proliferation capacity with a calculated doubling time of approximately 23.5 h in this 3D model (Supplementary Fig. 5D). Moreover, we screened several cell surface antigens by flow cytometry to characterize the protein expression levels of relevant markers (Supplementary Fig. 6). The negativity of CD45^+^ and FAP^+^ cells indicates the absence of hematopoietic and fibroblast-like cells. Also, the epithelial marker EpCAM stained lowly positive (Fig. 2E). Antigens that are often upregulated in cervical cancers, including the hyaluronic acid receptor CD44, its variant CD44v6, and Mesothelin, were either not detected or detected at low expression levels on isolated CeCa-5 cells. CD133, an antigen often associated with cancer stem cells, was detected on approximately 6% of cells. In contrast, a high expression was detected for MUC1 (± 80%) and the immune checkpoint protein PD-L1 (40%) (Fig. 2E).

### Genomic characterization

**Fig. 3:**
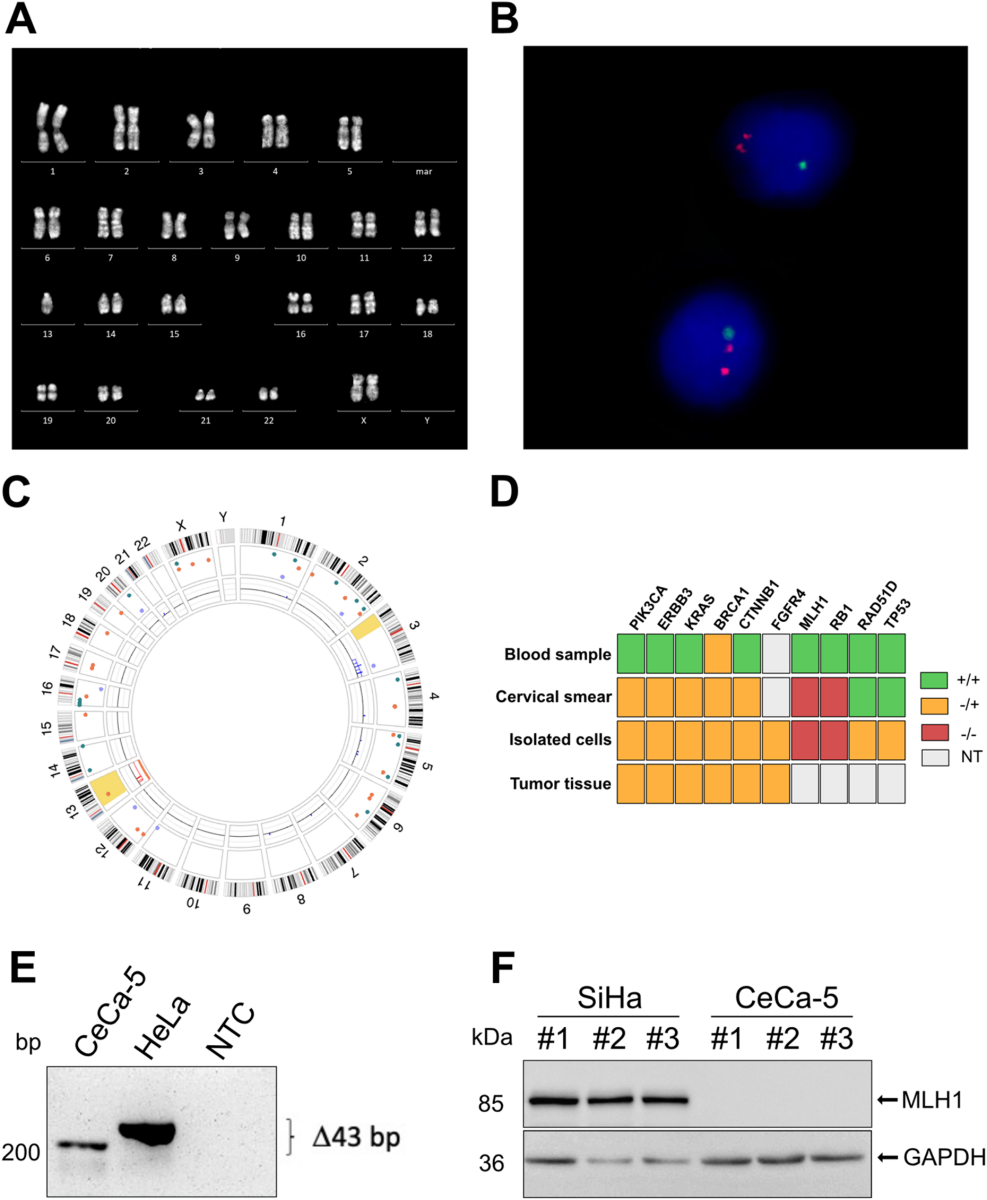
In-depth genetic phenotyping of CeCa-5 cells. The karyotype of CeCa-5 cells depicts the metaphase chromosomes of CeCa-5 cells (**A**). Loss of chromosome 13 was also detected by fluorescence in situ hybridization (FISH) to stain for chromosome 13 (green) and chromosome 21 (red), which was used as a reference (**B**). Circos plot displays chromosomal aberrations in CeCa-5 cells that were identified via optical genome mapping. Yellow highlighted areas indicate loss of heterozygosity (LOH) regions. Colored dots indicate the following variants: green color shaded circle-Insertion, orange color shaded circle-Deletion, blue color shaded circle-Inversion, and purple color shaded circle-Duplication (**C**). Panel of mutations identified by NGS and targeted sequencing of the peripheral blood mononuclear cells, the cervical smear sample, CeCa-5 cells, and the primary tumor tissue. Mutation status is displayed as follows: wild-type sequence (+/+) (green), heterozygous (-/+) (orange), and homozygous (-/-) (red) mutations, NT = not tested. Specimens not analyzed for mutations in the respective gene are shown in grey (**D**). Polyacrylamide gel electrophoresis of DNA amplicons was performed after *MLH1*-targeted amplification of cDNA prepared from RNA isolated from CeCa-5 and HeLa cells. The full-sized blot is depicted in Supplementary Fig. 9A. NTC = non-template control (**E**). Western blot of MLH1 and GAPDH using SiHa and CeCa-5 cells that were harvested at three different time points/passages (#1–3) (**F**). Full-sized Western blots are depicted in Supplementary Fig. 9C.

In-depth characterization of the genomic profile of CeCa-5 cells was performed to gain additional insights into tumor-driving aberrations present in the cells. Karyotyping revealed aneuploidy with the loss of chromosome 13 (monosomy 13) and duplication of 3q in CeCa-5 cells (45,XX,-13[6]/46,XX[11], nuc ish 3q26(EVI1 × 3)[11/100], 13q14.2(ITM2B/RB1/RCBTB2 × 1)[82/100]), which was supported by fluorescence in situ hybridization (FISH) (Fig. 3A and B, Supplementary Fig. 7). Optical genome mapping (OGM) was performed to identify structural variants and complex chromosomal rearrangements. Besides the confirmation of the chromosome 13 deletion and chromosome 3q duplication (present in the majority of the cells), we identified several duplications on chromosomes 1, 4, 5, 7, 8, and 21, which were only present in a subset of 30–40% of the cells (ogm[GRCh38] 1q41(217733498_220197498)x3[0.3], 3q13.31q29(114046411_198230596)x3[0.7], 4q31.3q32.1(154171897_157298214)x3[0.4], 5q11.2q12.1(58373464_60372378)x3[0.3], 5q22.1q22.2(110888916_112939899)x3[0.3], 5q23.2(123059431_126260941)x3[0.3], 7q21.11(83566695_85630956)x3[0.3], 8q23.3q24.11(114295718_117383688)x3[0.3], 13p11.2q34(16004725_114352102)x1[1.0], 21q21.1(15718969_17030141)x3[0.4]) (Fig. 3C, Supplementary Fig. 8, Supplementary File 1).

Next-generation sequencing (NGS)-based whole exome sequencing (WES) was used to characterize CeCa-5 cells further and to get additional, comprehensive insights into potential single-base aberrations. This study revealed 30,436 validated genetic variants, of which 12,530 had a minor allele frequency (MAF) < 0.005. The SnpEff tool was used to annotate the genetic variants. 792 variants were classified as missense substitutions, and 237 were classified as high impact (truncating or splicing). Among the truncating and splice variants in cancer-relevant genes, two were found in an apparently homozygous state in the established CeCa-5 cell line: a nonsense variant p.Arg320Ter in *RB1*, encoding the retinoblastoma tumor suppressor^19^ and a donor splice site variant c.588 + 2T > C in *MLH1*, encoding a mismatch repair protein (Table 1). The pathogenic effect of the novel *MLH1* splice variant was confirmed at the transcript level where it was associated with the loss of exon 7 (Fig. 3E, Supplementary Fig. 9A, B). Moreover, MLH1 protein levels were not detectable in CeCa-5 cells by Western blotting (Fig. 3F, Supplementary Fig. 9C). A frameshift variant c.392delT in *RAD51D* encoding a homology-directed repair protein was identified, but this was present only in the heterozygous state. Among the missense variants in cancer-relevant genes, only those that were either categorized as pathogenic in ClinVar or predicted pathogenic by ESM1b were further analyzed. These included known variants in *TP53* (Leu257Pro)^20–22^, *ERBB3* (p.Cys331Tyr), *PIK3CA* (p.Arg93Trp)^23^ and *KRAS* (p.Gly13Asp)^24^(Fig. 3D; Table 1). An unclassified rare missense variant in *BRCA1* was also noted (p.Glu9Gln) and followed but not predicted to be pathogenic.

The NGS-based Oncomine Focus Assay was used to compare the genomic profile of CeCa-5 cells with those of the primary tissue sample. The hereby detected variants in *CTNNB1*, *PIK3CA*, *FGFR4*, *KRAS*, *ERBB3*, and *BRCA1* coincide with those detected in CeCa-5 cells through WES (Fig. 3D). WES did not detect the monoallelic FGFR4*c.1930del frameshift variant, but it was confirmed through Sanger sequencing. The *CTNNB1* (pSer37Pro) missense variant, also monoallelic, was not clearly predicted to be pathogenic, though it is a known and potentially activating hotspot variant in different cancer types^25,26^. The stage at which the identified variants with known or predicted pathogenicity occurred during tumor development in the patient was further investigated. Therefore, sequences from germline DNA from a blood sample of the patient, as well as genomic DNA obtained from a preoperatively acquired cervical smear sample, were compared. Except for the likely benign *BRCA1* missense variant, none of the above-mentioned variants were detected in the patient´s germline DNA. The cervical smear already showed most of the variants, albeit at different proportions (Fig. 3D; Table 1, Supplementary Fig. 10). Interestingly, the *MLH1* and *RB1* variants already appeared almost homozygous in the cervical smears. In contrast, the *TP53* and *RAD51D* variants were not yet present in the cervical smear sample, indicating a later event during tumor cell evolution.

**Table 1:** Summary of identified mutations with known or predicted pathogenicity.

gene	Gene variant			Predicted variant effect	Proportion of variant cells		
	Variant ID	Nucleotide change	Protein change		Blood	Cervical smear	CeCa-5
*BRCA1*	rs1567823437	NM_007294.4:c.25G > C	NP_009225.1:p.Glu9Gln	ESM1b: -2.37 (benign)	0.5	0.5	0.5
*ERBB3*	rs2136798162	NM_001982.4:c.992G > A	NP_001973.2:p.Cys331Tyr	ESM1b: -13.95	0	0.5	0.5
*KRAS*	rs112445441	NM_033360.4:c.38G > A	NP_004976.2:p.Gly13Asp	ESM1b: -9.80, ClinVar pathogenic	0	0.3	0.5
*MLH1*	rs587779024	NM_000249.4:c.588 + 2T > C	Exon 7 skipping	Splicing proven	0	0.9	1.0
*PIK3CA*	rs1724342112	NM_006218.4:c.277 C > T	NP_006209.2:p.Arg93Trp	ESM1b: -10.99	0	0.5	0.5
*RAD51D*	rs772605790	NM_002878.4:c.392del	NP_002869.3:p.Asn131fs	Truncation	0	0	0.5
*RB1*	rs121913300	NM_000321.3:c.958 C > T	NP_000312.2:p.Arg320Ter	Truncation	0	0.8	1.0
*TP53*	rs28934577	NM_000546.6:c.770T > C	NP_000537.3:p.Leu257Pro	ESM1b: -14.28	0	0	0.5

DNA was extracted from the patient’s blood sample, a cervical smear sample, and CeCa-5 cells. The DNA was sequenced using whole exome sequencing (WES) and Sanger sequencing for confirmation. Genetic variants, the predicted variant effect, and the proportion of variant cells are displayed.

Beyond WES data analysis to identify genetic variants, we also screened WES data for the alignment with over 400 human HPV types cataloged in the Papillomavirus Episteme (PaVe) database. This alignment indicated no integration of any HPV type into the exome of CeCa-5 cells and thus further supports the results of the Vision*Array*® assay (Supplementary Fig. 3) and the HPV-negative classification of this tumor cell line.

### Targeted inhibition of CeCa-5 cells by anti-cancer drugs

**Fig. 4:**
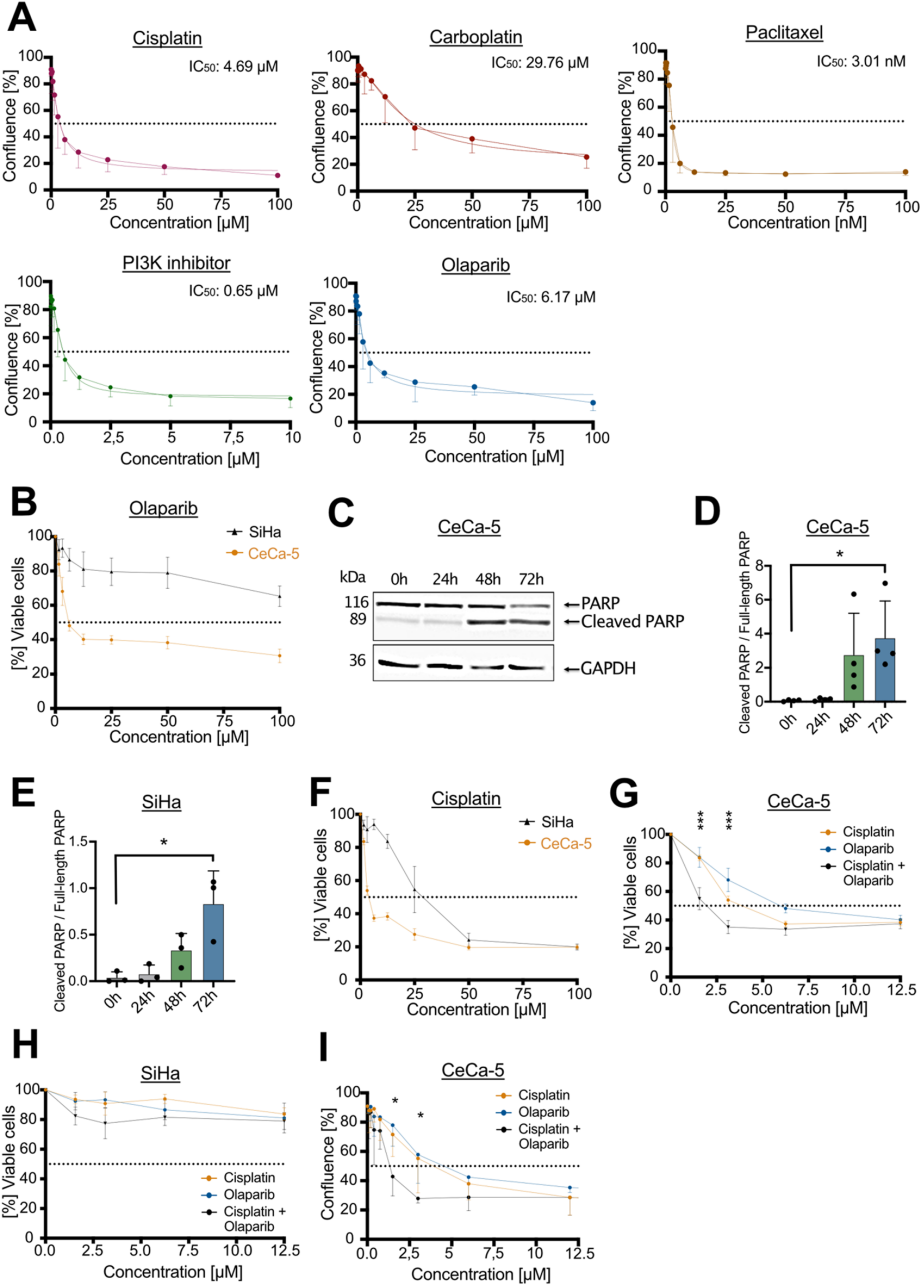
Functional evaluation of drug treatments against CeCa-5 cells. CeCa-5 cells were cultured in complete RPMI-1640 medium supplemented with multiple concentrations (0–10/100 µM/nM) of each drug to perform titrations using live-cell imaging. Cell confluence and the respective non-linear regression curves are depicted after 72 h of drug treatment (**A**). Flow-cytometric analysis of CeCa-5 and SiHa cells incubated with multiple olaparib concentrations (0–100 µM) (**B**). Western blots show GAPDH, PARP, and cleaved PARP signals after 0, 24, 48, and 72 h of olaparib treatment (6 µM) (**C**). PARP and cleaved PARP levels were normalized to GAPDH and the cleaved-to-full-length PARP ratio was quantified for CeCa-5 cells (**D**) and SiHa cells (**E**). Flow-cytometric analysis of CeCa-5 and SiHa cells incubated for 72 h with multiple cisplatin concentrations (0–100 µM) (**F**). CeCa-5 cells (**G**) and SiHa cells (**H**) were treated for 72 h with cisplatin, olaparib, or equimolar concentrations of both drugs in a combinatorial approach and analyzed via flow cytometry. Viable cells were identified as Annexin V and DAPI double negative (**B**, **F-H**). CeCa-5 cells were also treated with this combinatorial approach in a similar setup, but the cell confluence was analyzed via live-cell imaging (**I**). *n = 3–4.* Statistical significance is calculated in panels **G**-**I** for comparing cisplatin to cisplatin + olaparib groups and is denoted by *p ≤ 0.05, **p ≤ 0.01, ***p ≤ 0.001. Data are displayed as mean ± SD.

To functionally characterize CeCa-5 cells in more detail, we investigated the sensitivity of these cells to multiple chemotherapeutics and targeted drugs commonly used to treat cervical cancers. Therefore, CeCa-5 cells were incubated with the respective drugs at variable concentrations (0–100 µM) for 72 h, and their confluence was evaluated using live-cell imaging. As the NGS-based analysis revealed pathogenic variants with predicted functional impact in genes involved in DNA-damage pathways, we assessed the influence of cisplatin and carboplatin, which are known to induce DNA damage, on CeCa-5 cells. Here, CeCa-5 cells were susceptible to cisplatin (IC_50_=4.69 µM) and slightly less sensitive to carboplatin (IC_50_=29.76 µM) (Fig. 4A, Supplementary Fig. 11A). Compared to these platinum-based chemotherapeutics, CeCa-5 cells were more susceptible to the microtubule inhibitor paclitaxel (IC_50_=3.01 nM). Based on the detected variant in *PIK3CA* and the many genetic variants that were predicted to induce a sustained proliferative signal (Fig. 3D, E), we treated CeCa-5 cells with a PI3-Kα inhibitor 2 and identified an IC_50_ of 0.65 µM. Based on the chromosomal aberrations and the resulting haploinsufficiency for *BRCA2* and pathogenic variants of CeCa-5 cells identified in *MLH1* and *RAD51D* (Fig. 3E; Table 1), we also evaluated the PARP inhibitor olaparib and determined an IC_50_ value of 6.17 µM (Fig. 4A, Supplementary Fig. 11B). A similar IC_50_ value (IC_50_=6.2 µM) for olaparib was determined by analysis of the percentage of viable cells (Annexin V & DAPI double negative) after 72 h using flow cytometry. In contrast to CeCa-5, SiHa cells remained > 60% viable even at the highest tested concentration of 100 µM olaparib (Fig. 4B). Moreover, CeCa-5 cells revealed high ratios of cleaved to full-length PARP protein levels indicating apoptosis after 48 and 72 h of treatment with olaparib (Fig. 4C, D, Supplementary Fig. 12A). Cleaved PARP protein was also detected in SiHa cells that were treated with olaparib at the same concentration, albeit to a lower extent (Fig. 4E, Supplementary Fig. 12B). To investigate the potential combinatorial effects of drugs that induce DNA damage (e.g., platinum-based chemotherapeutics) with drugs that inhibit DNA damage repair (e.g., PARP inhibitors), CeCa-5 and SiHa cells were incubated with single drugs or with combinations of drugs. First, an IC_50_ value for cisplatin of 4.0 µM was assessed for CeCa-5 and 28 µM for SiHa cells using flow cytometry (Fig. 4F). By combining the treatment of cervical cancer cells using cisplatin with olaparib at equimolar concentrations, CeCa-5 cells revealed significantly less viable cells in the combinatorial setting (IC_50_=1.75 µM) as compared to the monotherapies (Fig. 4G). This combinatorial effect was not detected for SiHa cells (Fig. 4H). Besides flow cytometry, the benefit of the combinatorial treatment of CeCa-5 cells with cisplatin and olaparib was also detectable using live-cell imaging (IC_50_=1.61 µM) (Fig. 4I, Supplementary Fig. 13A). Combinations of olaparib with carboplatin, the PI3Kα2 inhibitor, and paclitaxel were explored based on the individual IC_50_ values for each drug. Here, solely the co-treatment of olaparib with carboplatin or cisplatin at lower concentrations provided a significant reduction compared to the monotherapy settings (Supplementary Fig. 13A–D).

## Discussion

### Need for novel models to study HPV-negative cervical cancer

Although HeLa, a cervical cancer cell line described in 1951^27^, constitutes the oldest described human cell line, the successful long-term culture of primary cervical cancer cells remains challenging. Since the detection of HPV DNA in cervical cancer cells by Dürst et al. (1983)^28^, cervical cancer has been categorized into HPV-positive and HPV-negative types. Over 90% of cervical cancers are classified as HPV-positive, while 3–8% are classified as HPV-negative^2^. Consequently, the majority of studies have been performed on HPV-positive cervical cancers, with HPV-negative types being largely underrepresented, e.g., less than 2% of cervical cancer studies that are published on PubMed include HPV-negative cervical cancers^12^. Today, only a few cervical cancer cell lines are considered HPV-negative, including C33A cells^13^ and OMC-4 cells^14^. As our understanding of HPV-negative cancers continues to lag behind that of HPV-positive cervical cancers, there is a clear need for new models to study this complex disease. The HPV-negative status of CeCa-5 cells described here was supported by screening for over 400 HPV types by VisionArray® Chip and WES analyses. Although the exome represents only a fraction of the entire genome, previous studies have demonstrated that HPV shows more integrations into exomes than predicted by chance, indicating a preference for integration into these regions^29^.

### Tumor microenvironment of the cervical cancer model

Compared to non-cancerous cervical tissues, cervical cancer tissues were reported to have high levels of fibroblast markers, including FAP^30^, which was also highly abundant in the primary tissue sample that was examined in this study (Fig. 1B, Supplementary Fig. 1, 2). The immune cell landscape, including the abundance and subtypes of immune cells, can vary strongly between different malignancies and can correlate with patient prognosis^31^. For example, CD163^+^ M2 macrophages were correlated with higher PD-L1 levels and poor prognosis in cervical cancer patients^32^. Here, CeCa-5 cells showed positivity for PD-L1, which was also detected in the primary tissue sample (Figs. 1A and 2E) and, therefore, might indicate sensitivity to checkpoint inhibitor-based therapies like pembrolizumab^33^.

The HPV-negative cervical cancer tissue from which CeCa-5 cells are derived showed comparable, but slightly lower, infiltration of immune cells (± 7.6% CD45^+^ cells) (Fig. 1B, Supplementary Fig. 1, 2) compared to HPV-positive cervical cancer specimens (average of 9.1% CD45^+^ cells) that our group previously prepared and examined with the same methods^34^. The infiltration and activation of immune cells within the cancer tissue might also be influenced by the HPV status of the patient, as HPV-negative cervical cancers revealed lower expression of MHC-I and -II compared to HPV-positive cervical cancers, thereby indicating reduced antigen presentation^35^. Of note, WES analysis revealed that CeCa-5 cells harbor 3 non-synonymous variants with MAF < 0.005 in HLA-A (Supplementary File 2), and two of them (rs1136741, rs1059563) have been previously predicted as probably damaging^36^. In fact, genetic variants in the HLA were previously identified as susceptibility loci for the development of cervical cancer^37^. Genetic variants that impair HLA expression and antigen presentation may be particularly critical in HPV-negative cervical cancers, as these tumors lack HPV-derived oncoproteins (such as E5 and E7) that actively suppress MHC-I surface expression in HPV-positive cancers^38,39^. This distinction suggests that HPV-negative cervical cancers may develop alternative immune escape mechanisms, including somatic mutations in MHC genes, to evade immune surveillance. However, given the limited number of studies investigating immune cell infiltration and immune evasion mechanisms in HPV-negative cervical cancers, further research is essential to gain a more comprehensive understanding of the tumor and its immune microenvironment in this cancer subtype.

### Genetic characterization of CeCa-5 cells

Cervical cancers are frequently associated with a duplication of chromosome 3q, which was reported to drive the development of cervical dysplasia to invasive cervical cancer^40^, and was also detected in CeCa-5 cells (Fig. 3C, Supplementary Fig. 7). While the oncogenic drivers of HPV-positive cervical cancer, including the viral oncoproteins E6 and E7, are well understood, the oncogenic drivers of HPV-negative cervical cancers are more diverse and associated with additional mutations, frequently affecting KRAS and p53 pathways^12,41^. In particular, mutations in *TP53* are typical for HPV-negative cervical cancers but are rarely detected in HPV-positive types due to the inherent function of the HPV oncoprotein E6, which promotes the degradation of p53 and p73^42^. Similarly, the HPV oncoprotein E7 can initiate the degradation of RB1 ^7^. Interestingly, CeCa-5 cells carry pathogenic mutations in *TP53* and *RB1* and thereby downregulate crucial tumor suppressor pathways without the need for HPV-associated oncoproteins (Fig. 3E; Table 1). As we identified a deletion of chromosome 13, which was also reported in the HPV-negative cancer cell line C33A^43^, on which *RB1* is encoded, the nonsense variant in *RB1* may in fact be hemizygous due to loss of heterozygosity, and this was an early event. Loss-of-function in such cell cycle regulators, together with the loss of mismatch repair due to the pathogenic *MLH1* splicing variant, may have triggered genome instability and led to further mutational events in relevant genes such as the *RAD51D* frameshift variant. Like the *RB1* nonsense variant, the *MLH1* splice site variant was an early mutational event as it already appeared almost homozygous in the cervical smear sample, although it was not present in the germline and the patient had no family history of Lynch Syndrome. However, this splice variant was predicted to be detrimental using MaxEntScan, and it was confirmed to result in exon skipping and the loss of MLH1 protein in the CeCa-5 line. The detected duplication of chromosome 3q, on which *PIK3CA* is located and encodes the catalytic subunit of PI3-kinase, is also of interest as this protein is commonly activated in cervical cancer. The identified missense variant p.Arg93Trp may further add to this activation (Table 1, Supplementary File 2), with *PI3KCA* variants being frequently observed in HPV-negative cervical cancer^44^. Additionally, CeCa-5 cells harbor mutations in *KRAS*, *PTEN*, *ARID5B*, *CTNNB1*, and *CCND1*, which were reported to be enriched in HPV-negative cervical cancers compared to HPV-positive tumors^45,46^. While approximately 96% of HPV-positive cervical cancers were detected to be positive for p16 by IHC staining, only 57% of HPV-negative cervical cancers were found to be p16-positive^8^, which is in line with the low, patchy p16 staining that was identified in the HPV-negative cancer tissue of this study (Fig. 1A, Supplementary Table 2). Moreover, NGS analysis revealed mutations in the *TERT* promoter (rs3215401, rs2853669) (Supplementary File 2), which were described to modify promoter activity and classified as risk variants^47,48^, potentially contributing to the high proliferation rate (Fig. 2C, Supplementary Fig. 5A, D) and immortalization of CeCa-5 cells.

The combination of these genetic aberrations that target distinct hallmarks of cancer presumably contributed to the transformation of CeCa-5 cells. Although we cannot exclude that a few variants such as those in *TP53* and *RAD51D* may have occurred during the cell culture propagation, the CeCa-5 model is likely to be a good representation of cervical cells from the primary tumor, as most likely causal variants that were identified in CeCa-5 were already detected in the cervical smear. None of them (except for one likely benign *BRCA1* variant) was present in the peripheral blood sample from this patient, consistent with no family history of cancer in her blood relatives.

### Targeted treatment strategies for HPV-negative cervical cancer

The results from genetic analyses of CeCa-5 cells revealed variants in genes implicated in homology-directed DNA double-strand break repair (HDR), such as a frameshift variant in *RAD51D* and a large deletion spanning *BRCA2*. Cancers with mutations in HDR genes have shown increased reliance on PARP for DNA repair, leading to a vulnerability of these cancers to PARP inhibitors, such as olaparib^49^. Based on these data, we evaluated the effects of olaparib on CeCa-5 cells and indeed observed a high sensitivity of CeCa-5 cells to this medication (Fig. 4A–D, Supplementary Fig. 12A). To further exploit defects in the DNA damage repair in a targeted treatment strategy, multiple studies reported promising results by combining PARP inhibitors with DNA-damage-inducing chemotherapeutics^50^, which is in line with the observations of this study, as both cisplatin and carboplatin showed enhanced cytotoxicity against CeCa-5 cells if they were given in combination with olaparib (Fig. 4G, I, Supplementary Fig. 13A–B). With regard to the clinical course, the patient was treated with radiochemotherapy using a total dose of 45 Gy (single dose = 1.8 Gy) for the pelvic and paraaortic lymph vessels, 50 Gy (single dose = 2.0 Gy) for the vaginal vault/uterine lodge, and 40 mg/m^2^ cisplatin weekly after surgery, but presented progressing residual cancer in the lymph nodes a few months later. In comparison to *BRCA1/BRCA2*-proficient SiHa cells, CeCa-5 cells were more sensitive to olaparib and the combination with chemotherapeutics (Fig. 4B, H), indicating that the effects of olaparib on CeCa-5 cells could be due to genetic aberrations that affect HDR. To contextualize the identified IC_50_ values, previous studies have reported IC_50_ values for olaparib in cervical cancer cell lines (SiHa, CaSki, ME180) ranging from 9 to 40 µM^51,52^, and for primary cervical cancer samples from 0.5 to 30 µM^53^. CeCa-5 cells might be particularly vulnerable to olaparib due to the detected mutations in MMR genes, here specifically *MLH1*, as cancer patients with defects in MMR have been reported to benefit from olaparib, especially when given in combination with other chemotherapeutics^54^.

Overall, we established a new HPV-negative cervical cancer cell line that maintained the genetic abnormalities found in the diagnostic presurgical sample even after isolation and passaging and maintained a high proliferative capacity in 2D and 3D models. Extensive genetic profiling, including broad HPV screening, karyotyping, FISH, OGM, and NGS analyses provided insights into the molecular evolution and characteristics of this carcinoma and make CeCa-5 cells a promising new resource to study the features and vulnerabilities of HPV-negative cervical cancers.

## Supplementary Information

Below is the link to the electronic supplementary material.

Supplementary Material 1

Supplementary Material 2

Supplementary Material 3

## Data Availability

The raw data supporting the conclusions of this article will be made available by the authors without undue reservation. The WES datasets generated and analyzed in this study are available at the European Nucleotide Archive (ENA) under the accession number ERR17278496.
