# Supplementary material for: Genomic profiling identifies actionable DNA-repair defects in a new cervical cancer model
Source: Sci Rep. 2026 Jul 29;16:23557. doi: 10.1038/s41598-026-59964-z (PMC13421491; doi:10.1038/s41598-026-59964-z)
Supplement: Supplementary file 3 — Supplementary Material 3 [file 41598_2026_59964_MOESM3_ESM.docx]

**Supplementary Material**

| Primer | Sequence |
| --- | --- |
| BRCA1_F | 5´- TGT GCT GAC TTA CCA GAT GG -3´ |
| BRCA1_R | 5´- GAC GTT GTC ATT AGT TCT TTG G -3´ |
| ERBB4_F | 5´- TCT GTC ACA GTG GAT TCG AG -3´ |
| ERBB4_R | 5´- TTG CTT AGG GAG CAA TGG AC -3´ |
| KRAS_F | 5´- TCT GTA TCA AAG AAT GGT CCT G -3´ |
| KRAS_R | 5´- CAT TAC GAT ACA CGT CTG CAG -3´ |
| MLH1_F | 5´- TCT GTG TTT TGA TTC AGT CAC C -3´ |
| MLH1_R | 5´- GTG TCC TAA CAT CAG CTA CTG -3´ |
| PIK3CA_F | 5´- CTG CTT TGG GAC AAC CAT AC -3´ |
| PIK3CA_R | 5´- AGA AAG GGA CAA CAG TTA AGC -3´ |
| RAD51D_F | 5´- TCA TCC TGG GTT TTA GCC TG -3´ |
| RAD51D_R | 5´- CAG CGT ACT AAT GGG TTC AG -3´ |
| RB1_F | 5´- GCT GAG AGA TGT AAT GAC ATG -3´ |
| RB1_R | 5´- CTA AAG GTC ACT AAG CTA AAG AC -3´ |
| TP53_F | 5´- GTT AAG AGG TCC CAA AGC CAG -3´ |
| TP53_R | 5´- TGG GCC TGT GTT ATC TCC TAG -3´ |

**Supplementary Table 1**. Primer sequences that were used for targeted sequencing. Sanger sequencing was performed using the listed primers to validate genetic variants that have been detected by whole exome sequencing (WES).

| Antigen | Status |
| --- | --- |
| ALK1 | **-** |
| BEREP4 | **-** |
| Chromogranin | **-** |
| CD3 | **-** |
| CD30 | **-** |
| CD34 | **-** |
| CD45 | **-** |
| CK5/14 | **-** |
| CK7 | **-** |
| CK8/18 | **-** |
| CK19 | **-** |
| CK20 | **-** |
| E-Cadherin | **-** |
| ERG | **-** |
| GATA-3 | **-** |
| MelanA | **-** |
| panCK | **-** |
| SALL4 | **-** |
| SOX10 | **-** |
| Synaptophysin | **-** |
| S100 | **-** |
| TTF1 | **-** |
| KI-67 | **+** (80%) |
| PAX8 | **+/-** (5%) |
| PD-L1 | **+** (3% TPS, CPS = 4) |
| p16 | **+/-** (unclear) |
| p40 | **+** (90%) |
| p63 | **+** |

**Supplementary Table 2**. Characterization of primary cervical cancer tissue by IHC. Positive stainings are displayed as “+”, negative stainings as “-“, and unclear stainings as “+/-“. TPS = Tumor Proportion Score, CPS = Combined Positive Score.


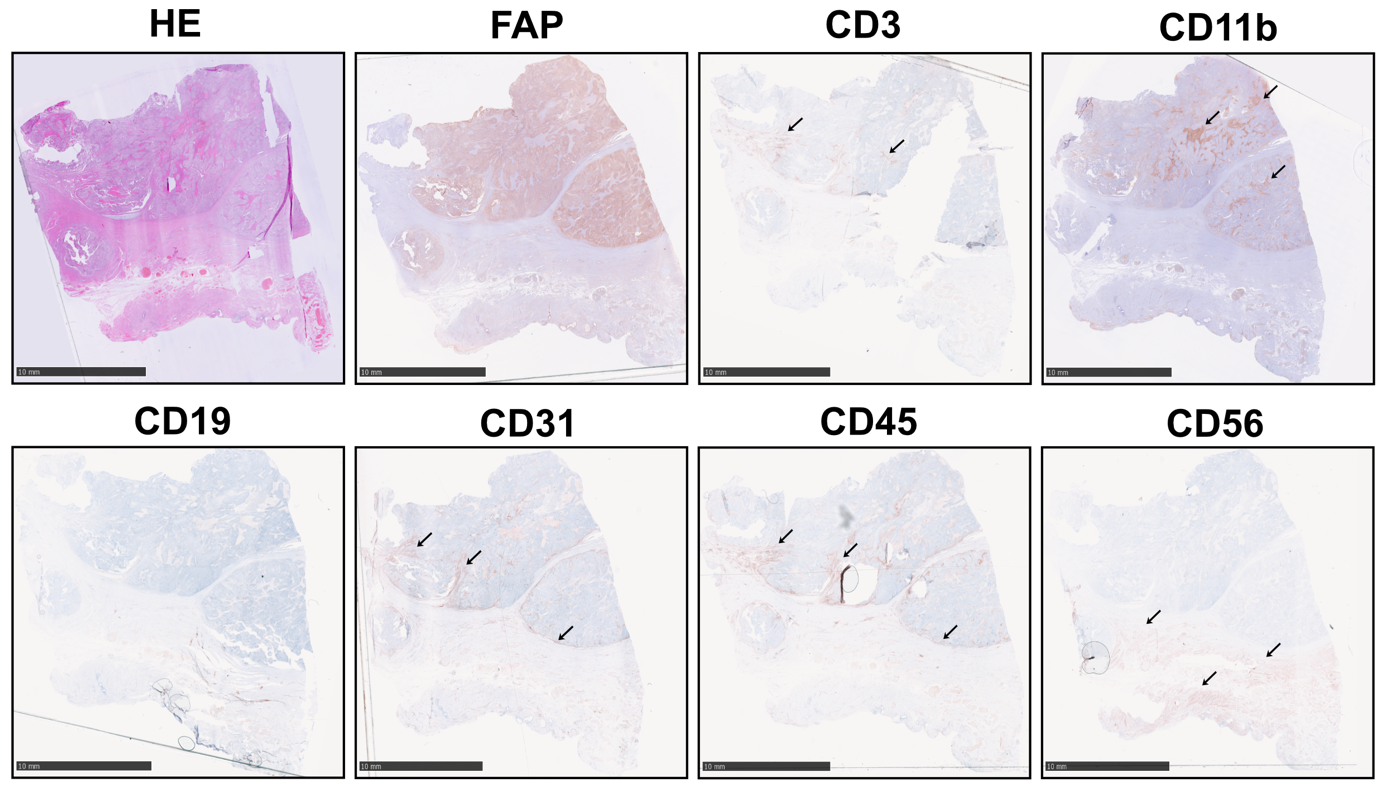


**Supplementary Figure 1**. Distribution of fibroblasts and immune cells in the primary cervical cancer tissue. Immunohistochemical staining was conducted on the primary cervical cancer tissue to investigate the tissue distribution of fibroblasts (FAP) and immune cells (CD3, CD11b, CD19, CD31, CD45, CD56). The images correspond to those depicted in **Figure 1B**, but are not magnified to enable an overview of the antigen-positive cell distribution in the tissue. Arrows indicate antigen-positive regions. Scale bars correspond to 10 mm.


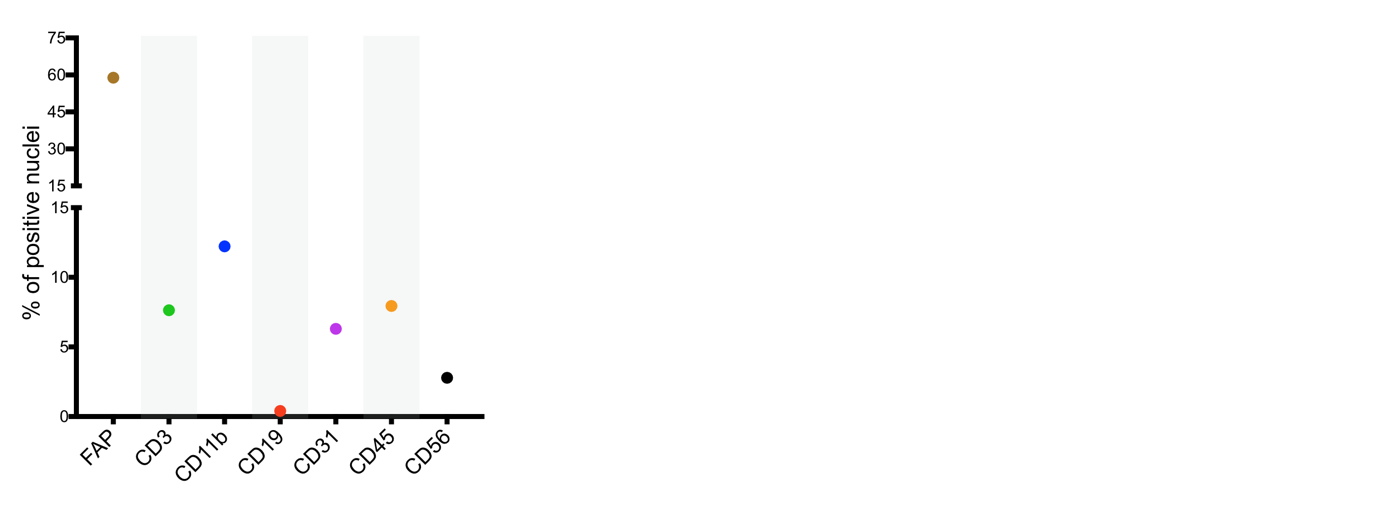


**Supplementary Figure 2**. Quantification of IHC stainings that are shown in **Figure 1B**. Stainings were performed to identify the presence of immune cells (CD3, CD11b, CD19, CD31, CD45, CD56) and fibroblasts (FAP) in the primary cervical cancer tissue, *n=1*.


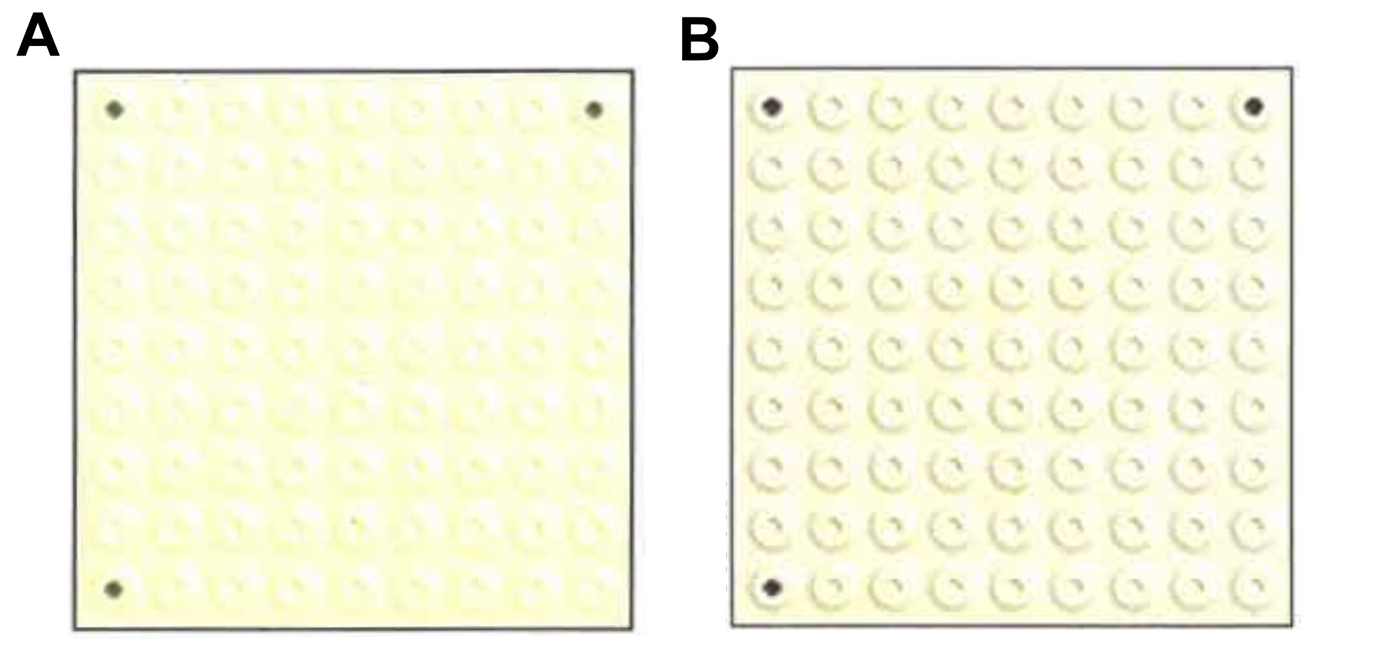


**Supplementary Figure 3**. HPV testing of cervical cancer. The primary cervical cancer tissue (**A**) and isolated CeCa-5 cells derived from this tissue (**B**) were screened for 41 clinically relevant HPV genotypes using the Vision*Array*^®^ Chip. Positive signals are depicted as black dots on the chip. The bottom left, top left, and top right wells serve as positive controls.


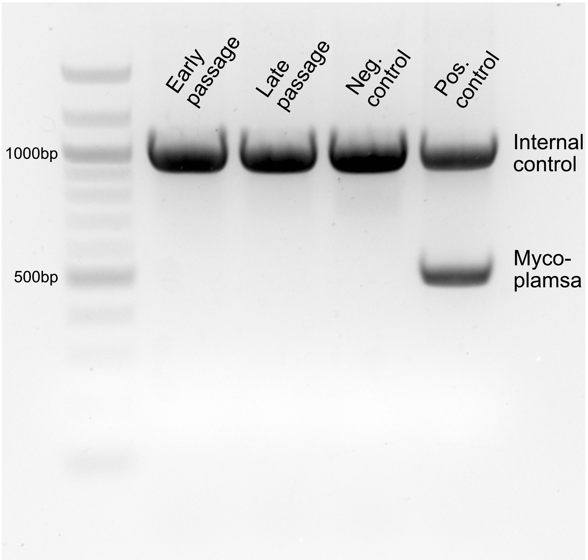


**Supplementary Figure 4**. The cervical cancer cells tested negative for mycoplasma contamination. Amplification of potential mycoplasma DNA was performed by PCR. Agarose gel electrophoresis of the amplified PCR amplicons shows the absence of mycoplasma in early and late passages of CeCa-5 cells.


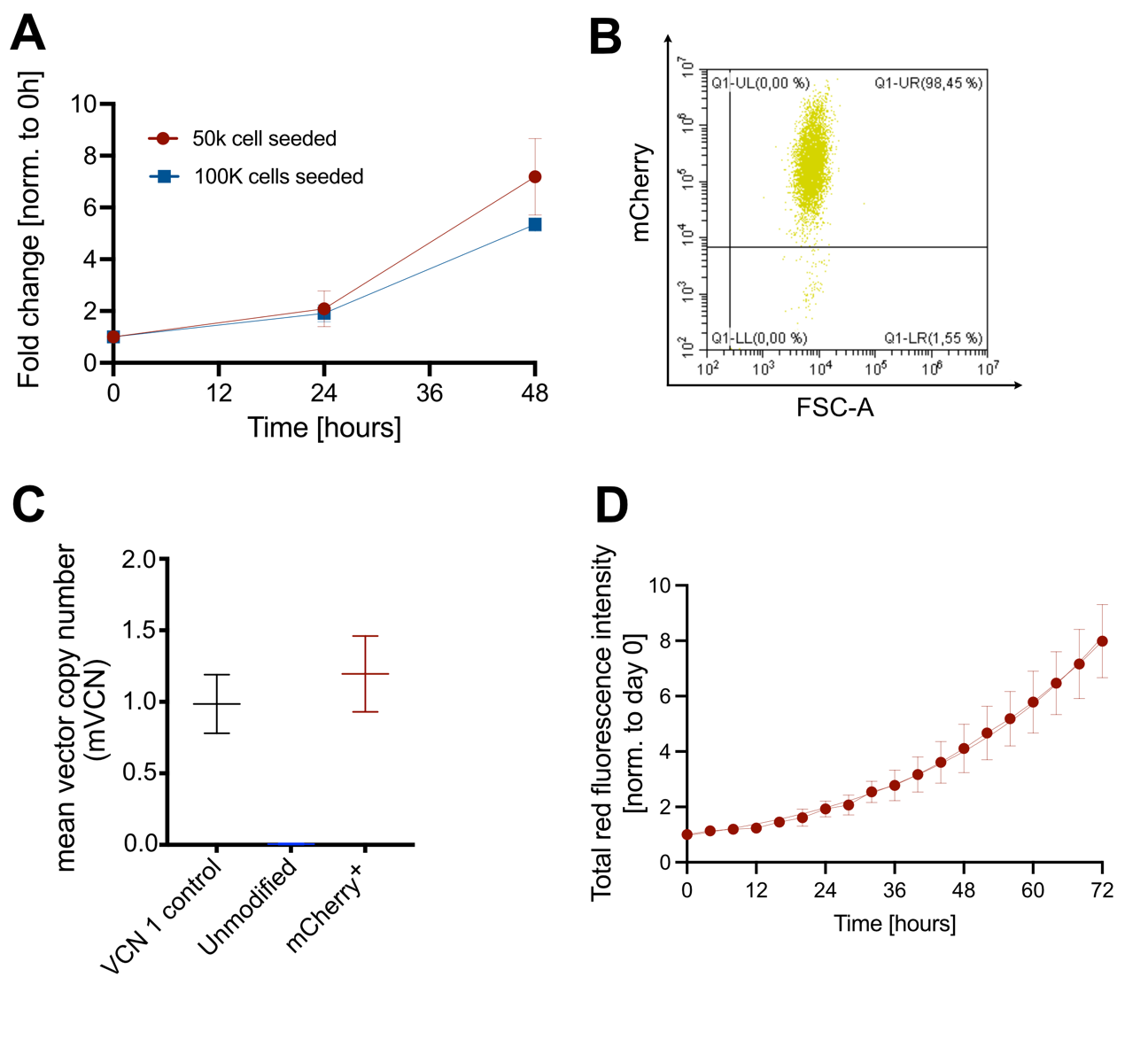


**Supplementary Figure 5**. Proliferation and vector copy number determination of mCherry^+^ CeCa-5 cells. The proliferation of 50,000 and 100,000 initially seeded CeCa-5 cells was analyzed by flow cytometry after 24 and 48 hours in a 2D model (**A**) (*n=3*). CeCa-5 cells were transduced with a lentiviral vector to stably express mCherry (**B**), their mean vector copy number (mVCN) was assessed (**C**) (*n=2*), and their red fluorescence intensity was used to quantify their proliferation every 4 hours in a 3D tumor spheroid model (**D**), *n=3.* Data are displayed as mean ± SD.


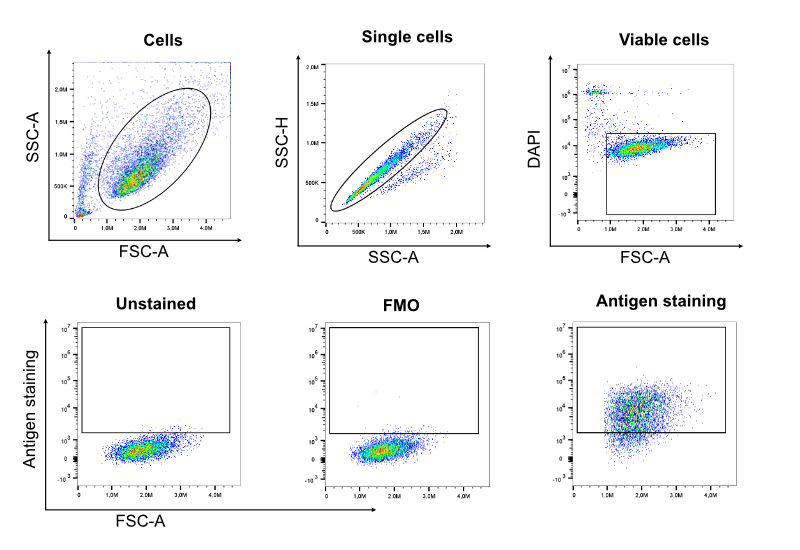


**Supplementary Figure 6**. Flow cytometric gating strategy to detect cell surface antigens. SSC-A & SSC-H identified single cells, and DAPI-negative cells were defined as viable cells. Gates were set based on the Fluorescence Minus One (FMO) sample.


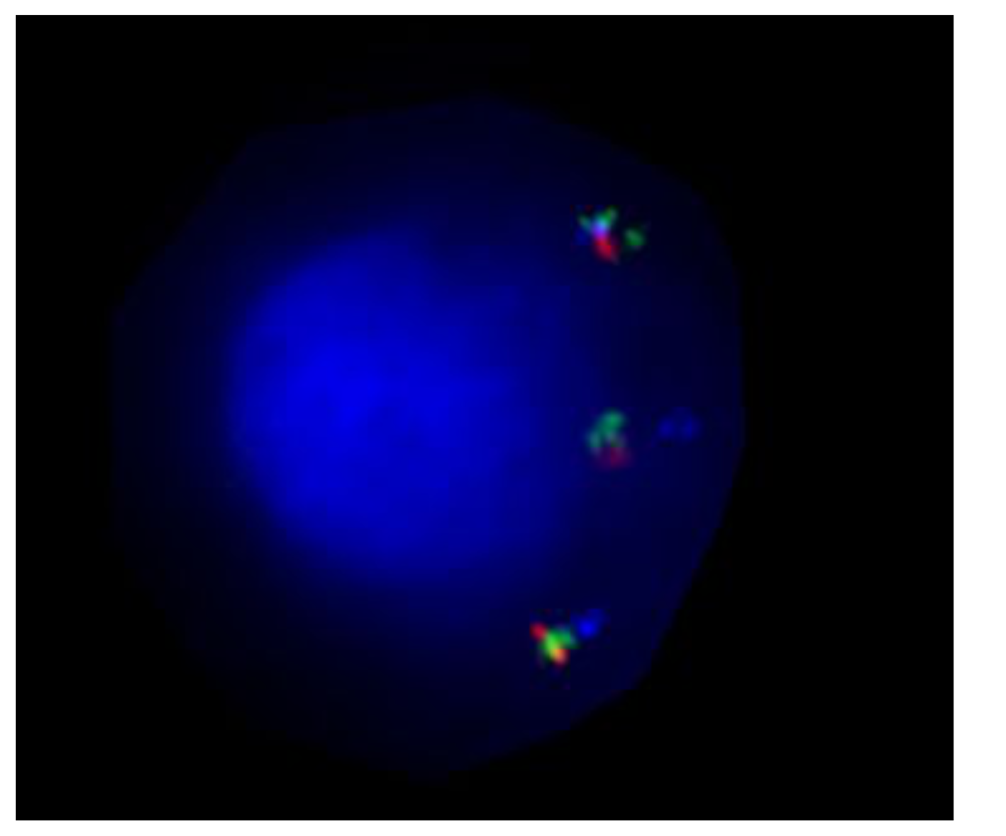


**Supplementary Figure 7.** FISH analysis of chromosome 3q.26. Duplication of chromosome 3q was detected by fluorescence *in situ* hybridization (FISH) analysis, showing staining of the GOLIM4/EGFEM1P (blue), EVI1 (MECOM) (green), and MYNN/LRRC34 (red) on chromosome 3q26.2.


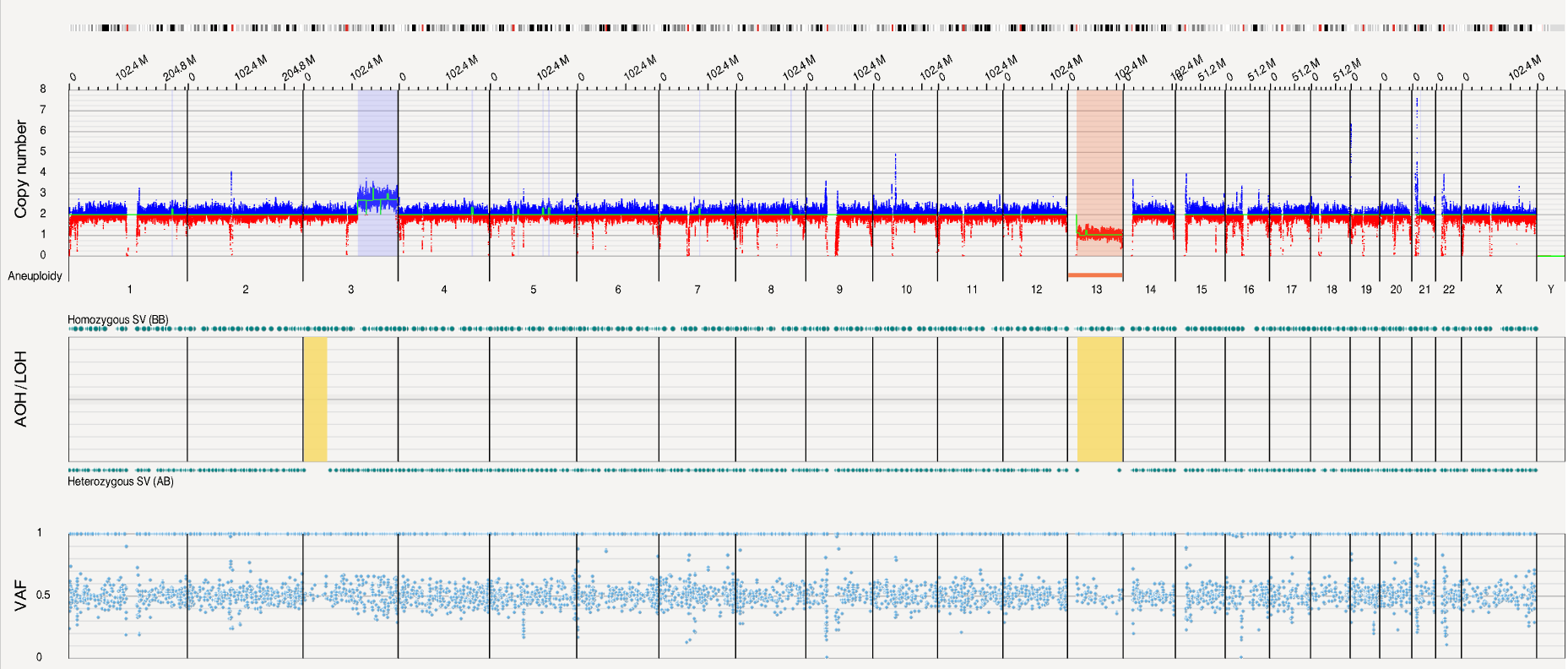


**Supplementary Figure 8.** Genomic profile of CeCa-5 cells as assessed via optical genome mapping. Optical genome mapping (OGM) was performed to identify potential chromosomal rearrangements and structural variants. Copy numbers, absence of heterozygosity (AOH)/loss of heterozygosity (LOH), and the variant allele frequencies (VAFs) are displayed. SV = structural variant.

**
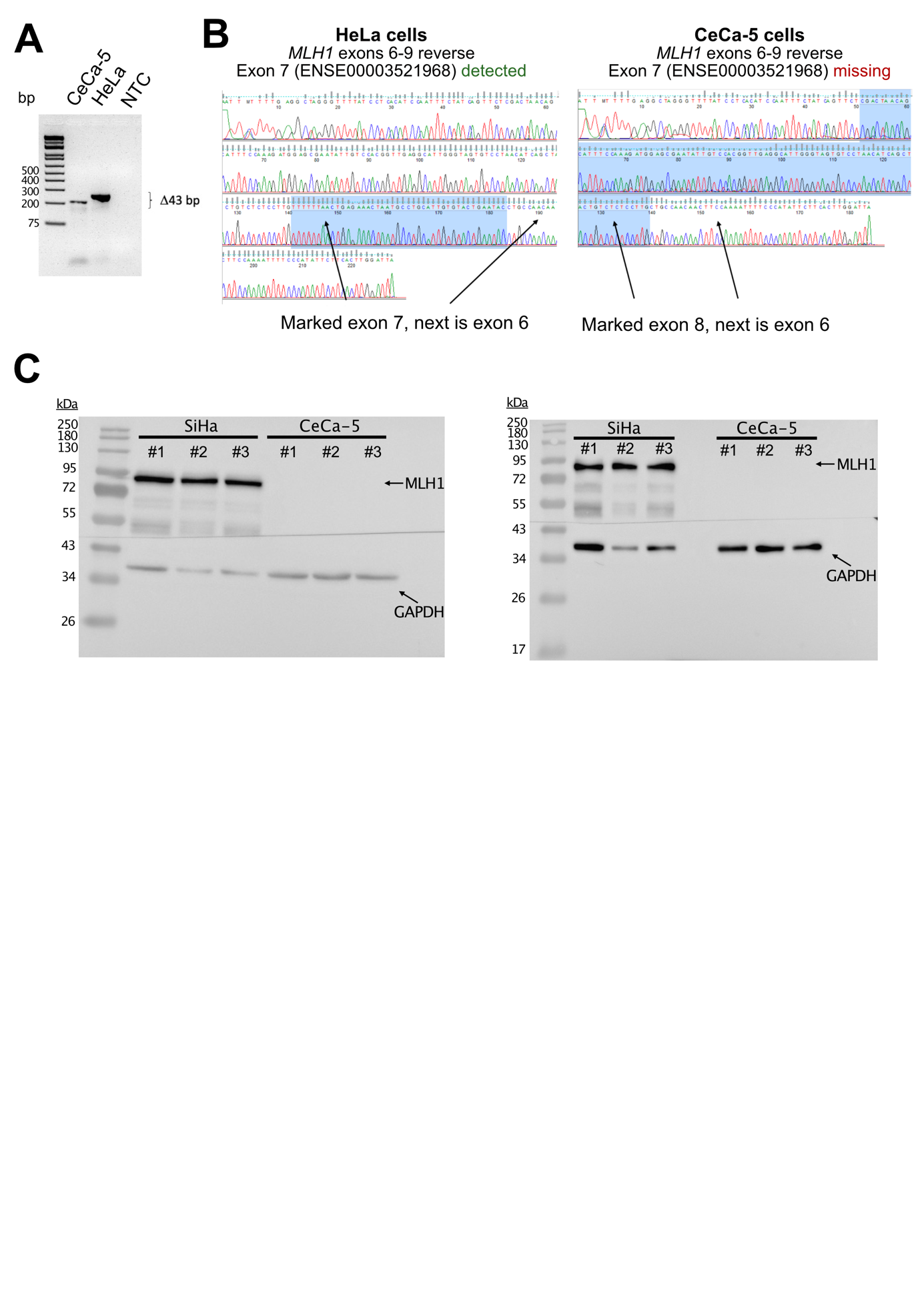
**

**Supplementary Figure 9.** Detection of MLH1 transcript and proteins in cervical cancer cells. Polyacrylamide gel electrophoresis of the amplified DNA was performed after amplifying the reversely transcribed MLH1 RNA in CeCa-5 and HeLa cells. NTC = non-template control (**A**). Sequencing of CeCa-5 and Hela cells highlights the absence of exon 7 in CeCa-5 cells (**B**). Western blotting of MLH1 and GAPDH using SiHa and CeCa-5 cells that were harvested at three different time points/passages and were repeated twice (#1-3) (**C**).


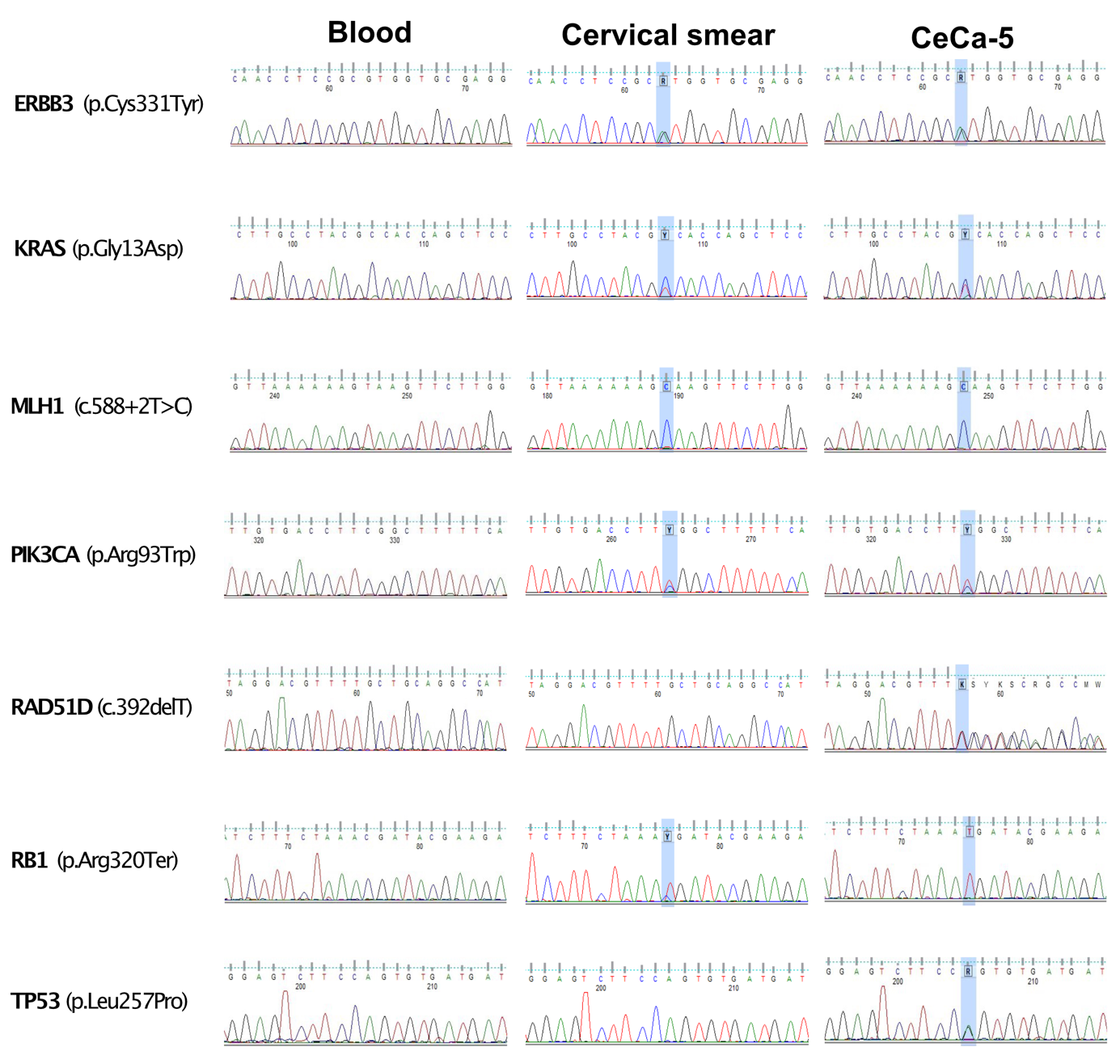


**Supplementary Figure 10**. Electropherograms of Sanger sequencing results for pathogenic variants discovered in the cervical tumor. DNA was extracted from the patient's blood sample, a cervical smear sample, and CeCa-5 cells and sequenced using Sanger sequencing.


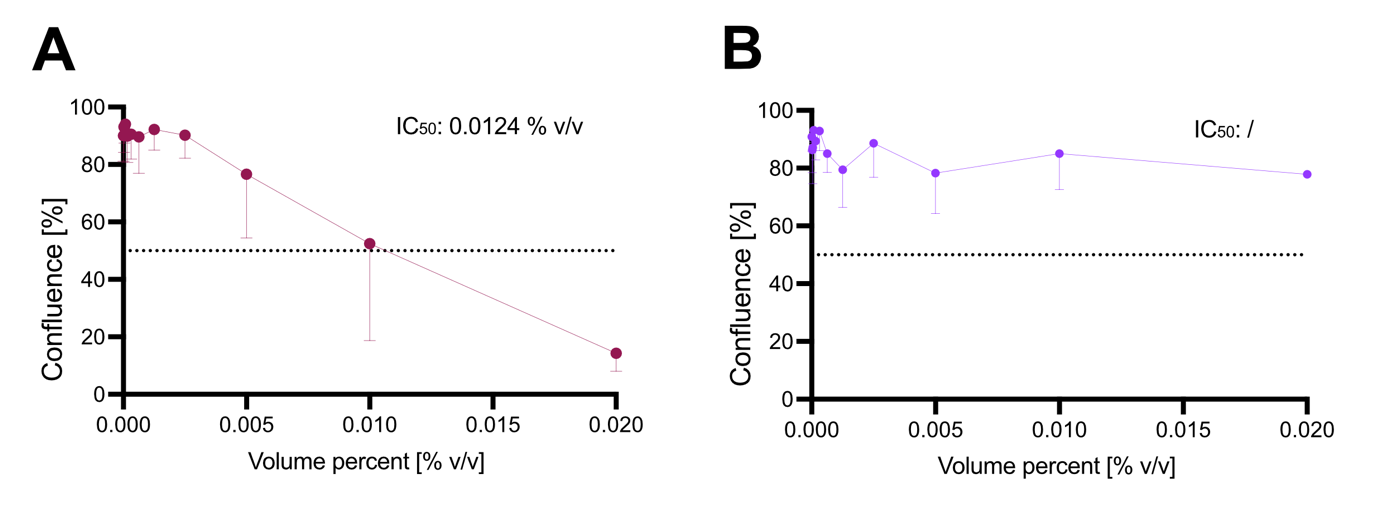


**Supplementary Figure 11.** Live-cell imaging of CeCa-5 cells that were treated with solvent controls. CeCa-5 cells were treated with the same volumes of DMSO (**A**) or NaCl (**B**) as the drugs tested in **Figure 4A** (*e.g.,* a drug that was dissolved in DMSO and used at a concentration of 100 µM, contained 0.02% v/v DMSO; 50 µM ≙ 0.01% v/v). *n=3.* Data are displayed as mean ± SD.


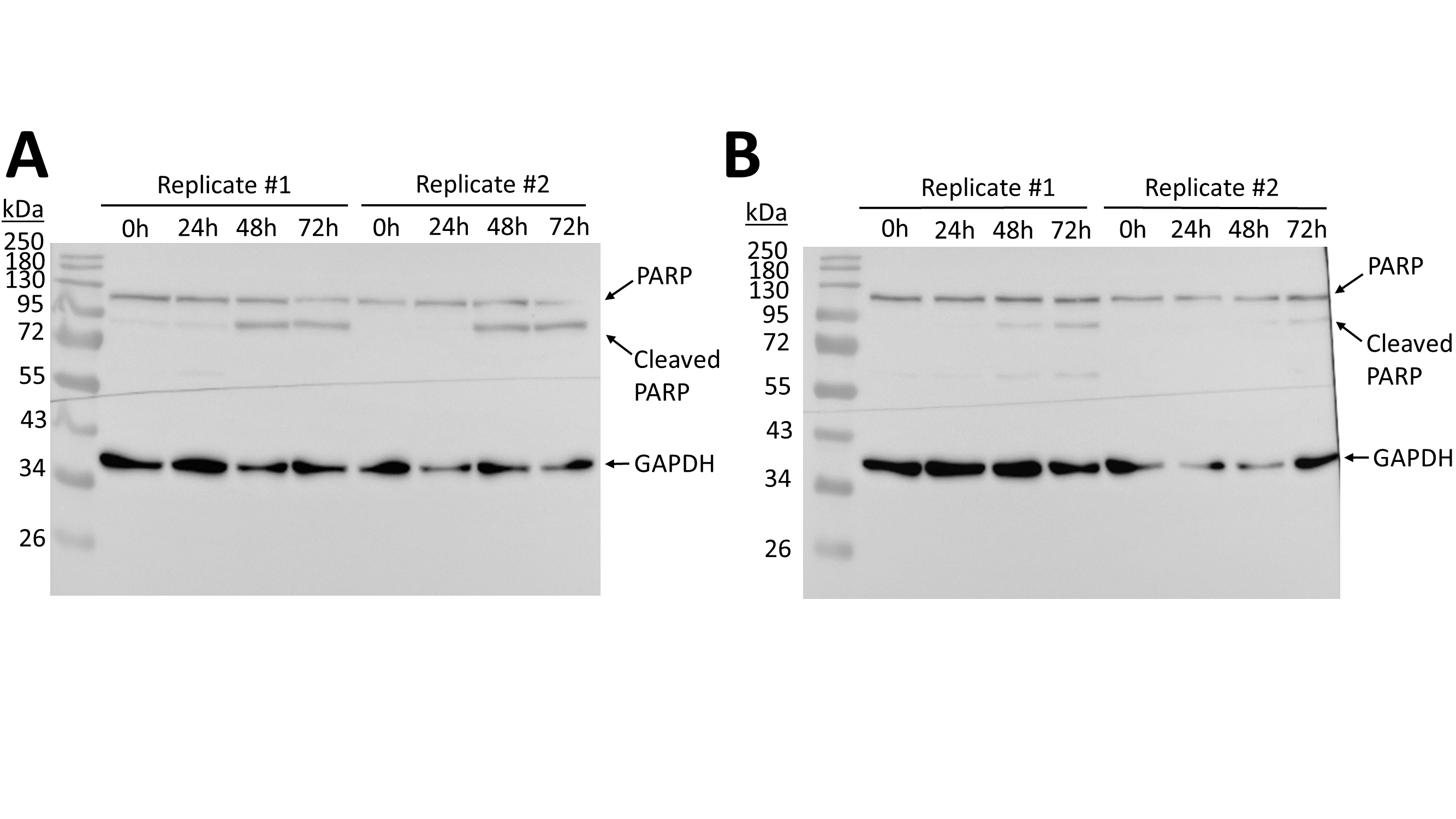


**Supplementary Figure 12**. Detection of PARP cleavage after olaparib treatment. Western blots show the detection of GAPDH, PARP, and cleaved PARP after 0h, 24h, 48h, and 72 hours of treatment of CeCa-5 cells (**A**) and SiHa cells (**B**) with 6 µM of olaparib. Before antibody staining, the membranes were cut in the center to enable separate antibody incubations. After staining, the two parts were rejoined and imaged simultaneously.


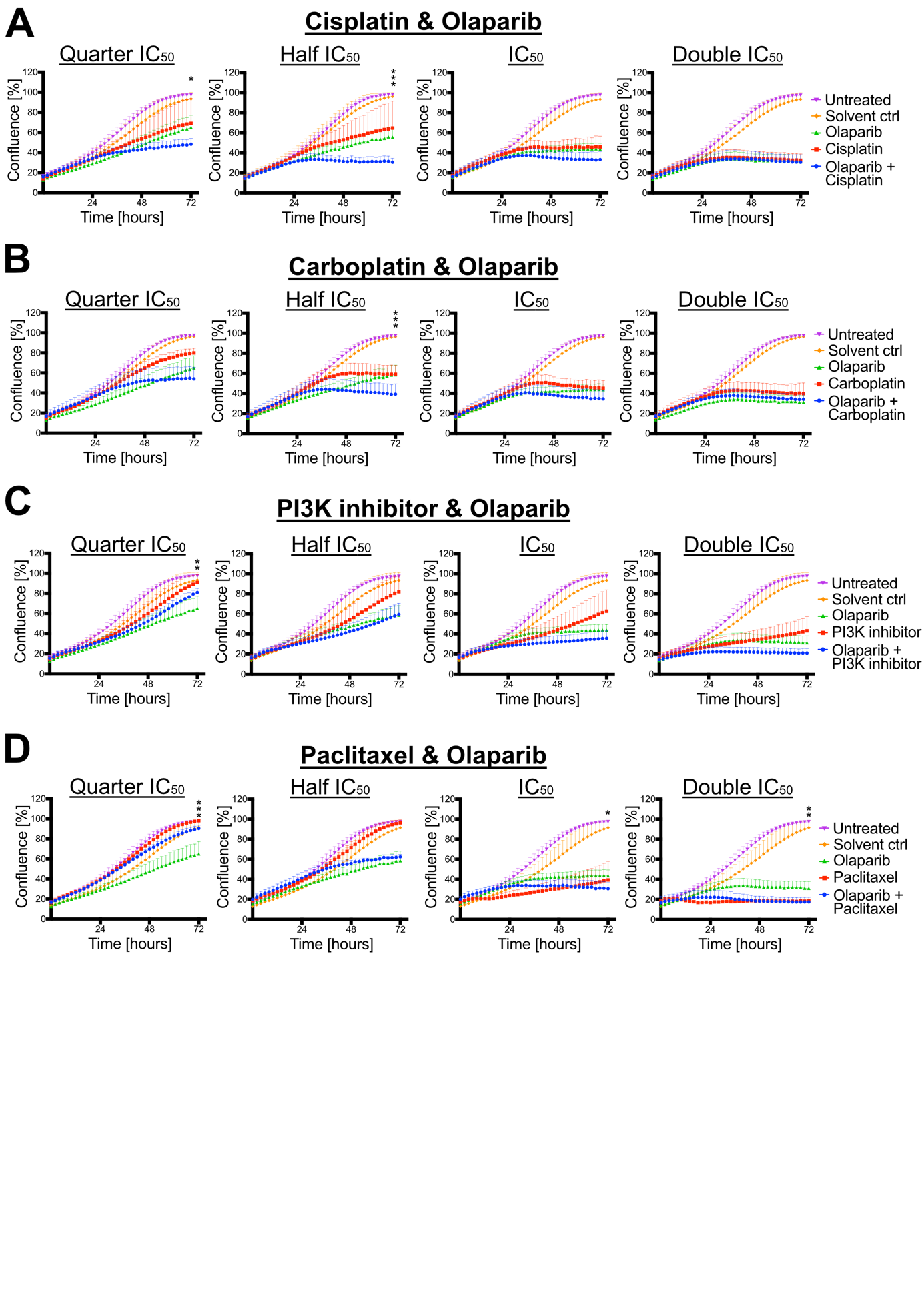


**Supplementary Figure 13.** Treatment of CeCa-5 cells with different drug combinations. Olaparib was combined with cisplatin (**A**), carboplatin (**B**), PI3K inhibitor (**C**), or paclitaxel (**D**). To detect the potential benefits of combinatorial treatments, the applied concentrations were based on the IC_50_ values of each drug and covered a range below and above the respective IC_50_ values (*n=2-4*). Statistical significance is calculated for comparing olaparib to cisplatin/carboplatin/PI3K inhibitor/paclitaxel + olaparib groups and is denoted by *p≤0.05, **p≤0.01, ***p≤0.001. Data are displayed as mean ± SD.
